# Comparative Genome Analysis of *Scutellaria baicalensis* and *Scutellaria barbata* Reveals the Evolution of Active Flavonoid Biosynthesis

**DOI:** 10.1016/j.gpb.2020.06.002

**Published:** 2020-11-04

**Authors:** Zhichao Xu, Ranran Gao, Xiangdong Pu, Rong Xu, Jiyong Wang, Sihao Zheng, Yan Zeng, Jun Chen, Chunnian He, Jingyuan Song

**Affiliations:** 1 Key Lab of Chinese Medicine Resources Conservation, State Administration of Traditional Chinese Medicine of China, Institute of Medicinal Plant Development, Chinese Academy of Medical Sciences & Peking Union Medical College, Beijing 100193, China; 2 Engineering Research Center of Chinese Medicine Resource, Ministry of Education, Beijing 100193, China; 3 China National Traditional Chinese Medicine Co., Ltd, Beijing 102600, China

**Keywords:** *Scutellaria*, Whole-genome duplication, Flavonoid biosynthesis, Tandem duplication, Species-specific evolution

## Abstract

*Scutellaria baicalensis* (*S. baicalensis*) and *Scutellaria barbata* (*S. barbata*) are common medicinal plants of the Lamiaceae family. Both produce specific flavonoid compounds, including baicalein, scutellarein, norwogonin, and wogonin, as well as their glycosides, which exhibit antioxidant and antitumor activities. Here, we report chromosome-level genome assemblies of *S. baicalensis* and *S. barbata* with quantitative chromosomal variation (2*n* = 18 and 2*n* = 26, respectively). The divergence of *S. baicalensis* and *S. barbata* occurred far earlier than previously reported, and a **whole-genome duplication** (WGD) event was identified. The insertion of long terminal repeat elements after speciation might be responsible for the observed chromosomal expansion and rearrangement. Comparative genome analysis of the congeneric species revealed the **species-specific evolution** of chrysin and apigenin biosynthetic genes, such as the *S. baicalensis*-specific **tandem duplication** of genes encoding phenylalanine ammonia lyase and chalcone synthase, and the *S. barbata*-specific duplication of genes encoding 4-CoA ligase. In addition, the paralogous duplication, colinearity, and expression diversity of *CYP82D* subfamily members revealed the functional divergence of genes encoding flavone hydroxylase between *S. baicalensis* and *S. barbata*. Analyzing these ***Scutellaria*** genomes reveals the common and species-specific evolution of flavone biosynthetic genes. Thus, these findings would facilitate the development of molecular breeding and studies of biosynthesis and regulation of bioactive compounds.

## Introduction

Plant-specific flavonoids, including flavones, flavonols, anthocyanins, proanthocyanidins, and isoflavones, play important functions in plants. These functions include flower pigmentation, ultraviolet protection, and symbiotic nitrogen fixation [1], [2], [3]. Flavonoid metabolites also have biological and pharmacological activities on human health, including antibacterial and antioxidant functions, and the treatment of cancer, inflammatory, and cardiovascular diseases [3]. The genus *Scutellaria*, which belongs to the Lamiaceae family, consists of common herbal plants enriched in bioactive flavonoids. Approximately 300–360 *Scutellaria* species have the characteristic flower form of upper and lower lips [4], [5]. Nonetheless, only two *Scutellaria* species, *S. baicalensis* and *S. barbata*, are recorded in the Pharmacopoeia of the People’s Republic of China. The roots of *S. baicalensis* and dried herbs of *S. barbata* are the basis of the traditional Chinese medicine (TCM) *Huang Qin* and *Ban Zhi Lian*, respectively, which have been used as heat-clearing and detoxifying herbs for thousands of years [6]. The main biologically active compounds in *Scutellaria* are derivatives of chrysin and apigenin, such as baicalein, scutellarein, and wogonin, as well as their glycosides, which include baicalin, scutellarin, and wogonoside [7], [8], [9], [10]. The demonstration that baicalin activates carnitine palmitoyltransferase 1 in the treatment of diet-induced obesity and hepatic steatosis [11], [12] has generated extensive interest in the potential antilipemic effect of this compound.

Illuminating the chemodiversity and biosynthesis of the active constituents of *Scutellaria* will provide a foundation for investigating the use of *Huang Qin* and *Ban Zhi Lian* in TCM, and the production of these natural products via synthetic biology [13]. In *S. baicalensis*, the biosynthetic genes of the root-specific compounds baicalein and norwogonin have been functionally identified, providing an important basis for studies of the biosynthesis and regulation of the natural products [14], [15]. Recently, the *in vivo* production of baicalein and scutellarein in *Escherichia coli* and *Saccharomyces cerevisiae* was achieved based on the guidance of synthetic biology [16], [17]. However, the discovery and optimization of biological components are important limitations to the metabolic engineering of these compounds. *Salvia miltiorrhiza* (Lamiaceae family) genome has provided useful information on secondary metabolism for the rapid functional identification of biosynthetic and regulatory genes [18], [19], [20], [21], [22], [23]. In contrast, the genomes of the *Scutellaria* genus remain unclear, and the reliance on transcriptome data from short-read sequencing has restricted gene discovery and analyses of genome evolution, including studies of gene family expansion and contraction, evolution of biosynthetic genes, and identification of regulatory elements [24].

**Figure 1:**
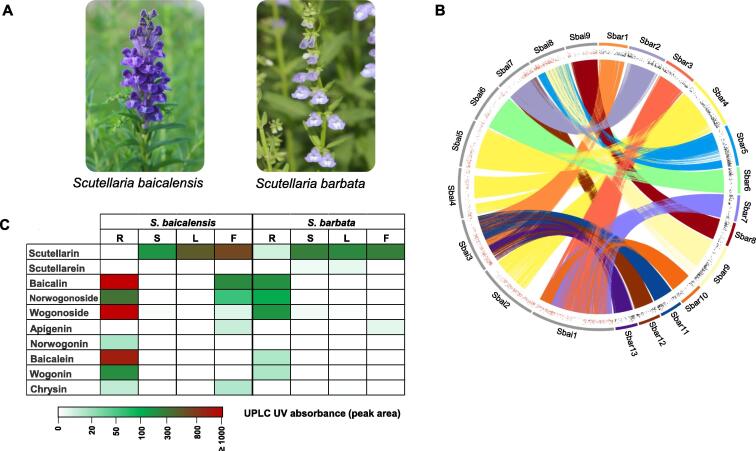
**Genome colinearity reveals the chromosome rearrangement in *Scutellaria*** **A.** Morphological differences between the aerial parts of *S. baicalensis* and *S. barbata*. **B.** Comparison of nucleotide sequences of 9 *S. baicalensis* chromosomes (Sbai1–Sbai9; gray bars) and 13 *S. barbata* chromosomes (Sbar1–Sbar13; colored bars). Mapped regions with > 90% sequence similarity over 5 kb between the two species were linked. The red and black dots represent up-regulated genes (Log_2_ FC > 1, FPKM > 10) in the root tissue compared to other tissues (stem, leaf, and flower) in *S. baicalensis* and *S. barbata*, respectively. **C.** Content distribution of flavone compounds in different tissues of *S. baicalensis* and *S. barbata*, including root, stem, leave, and flower, determined by UPLC. FC, fold change; FPKM, fragments per kilobase of exon model per million reads mapped; UPLC, ultraperformance liquid chromatography; R, root; S, stem; L, leaf; F, flower.

Morphological differences are present at the macroscopic level between *S. baicalensis* and *S. barbata*. Differentiation of these species are characterized mainly by the fleshy rhizome and branched stem of *S. baicalensis* and the fibrous root and erect stem of *S. barbata* (**Figure 1**A). The active compounds baicalein, wogonin, and scutellarein are differentially distributed in the roots and aerial parts of *S. baicalensis* and *S. barbata*. Here, we performed *de novo* sequencing and assembly of the *S. baicalensis* and *S. barbata* genomes using a long-read strategy and high-through chromosome conformation capture (Hi-C) technology. The chromosome-level genomes of *S. baicalensis* and *S. barbata* revealed their divergence time, chromosomal rearrangement and expansion, whole-genome duplication (WGD), and evolutionary diversity of flavonoid biosynthesis. The data provided important insights for the molecular assisted breeding of important TCM resources, genome editing, and increased understanding of the molecular mechanisms of the chemodiversity of active compounds.

## Results and discussion

### High-quality assembly of two *Scutellaria* genomes

The size of the *S. baicalensis* genome was predicted to be 440.2 ± 10 Mb and 441.9 Mb by using the flow cytometry and the 21 *k*-mer distribution analysis (approximately 0.96% heterozygosity), respectively (Figure S1). The genome survey of *S. barbata* revealed a 404.6 Mb genome size and 0.28% heterozygosity via the 21 *k*-mer distribution analysis (Figure S1). Third-generation sequencing platforms, including PacBio and Oxford Nanopore technologies, have been confirmed to have important advantages in *de novo* assembly and in processing data with complex structural variation due to high heterozygosity and repeat content [25], [26], [27]. Thus, 52.1 Gb Oxford Nanopore technology (ONT) reads (~120×) with an N50 of 16.3 kb from *S. baicalensis* and 51.7 Gb single molecule, real-time sequencing (SMRT) reads from the PacBio platform (~130×) with an N50 of 9.8 kb from *S. barbata* were produced to assemble highly contiguous genomes (Table S1). The low-quality long reads were further corrected and trimmed to yield 20.2 Gb ONT reads with an N50 of 35.5 kb from *S. baicalensis* and 18.0 Gb SMRT reads with an N50 of 15.3 kb from *S. barbata* using the Canu pipeline.

The contiguous assembly of the *S. baicalensis* and *S. barbata* genomes was performed using the optimized SMARTdenovo and 3× Pilon polishing (50× Illumina reads) packages. For *S. baicalensis*, the contig-level genome assembly, which was 377.0 Mb in length with an N50 of 2.1 Mb and a maximum contig length of 9.7 Mb, covered 85.3% of the estimated genome size (Table S2). The *S. baicalensis* genome identified 91.5% of the complete Benchmarking Universal Single-Copy Orthologs (BUSCO) gene models and had an 88.7% DNA mapping rate, suggesting a high-quality genome assembly. For *S. barbata*, the contiguous contig assembly of 353.0 Mb with an N50 of 2.5 Mb and a maximum contig of 10.5 Mb covered 87.2% of the predicted genome size (Table S2). The *S. barbata* genome identified 93.0% of the complete BUSCO gene models and had a 95.0% DNA mapping rate. The high-quality genome assemblies of *S. baicalensis* and *S. barbata* showed the great advantage of single molecule sequencing, with assembly metrics that were far better than those of other reported genomes of Lamiaceae species, *i.e.*, *S. miltiorrhiza* [22] and *Mentha longifolia* [28].

Given the assembly continuity, with a contig N50 of over 2 Mb for the *S. baicalensis* and *S. barbata* genomes, Hi-C technology was applied to construct chromosome-level genomes [29]. In total, 99.8% and 98.8% of the assembled *S. baicalensis* and *S. barbata* contigs were corrected and anchored to 9 and 13 pseudochromosomes (2*n* = 18 for *S. baicalensis*, 2*n* = 26 for *S. barbata*) using a Hi-C interaction matrix with N50 values of 40.8 Mb and 23.7 Mb, respectively. The strong signal along the diagonal of interactions between proximal regions suggested the high-quality of the Hi-C assemblies for the *S. baicalensis* and *S. barbata* genomes (Figure S2).

The *S. baicalensis* genome comprised 33,414 protein-coding genes and 2833 noncoding RNAs (ncRNA). For the *S. barbata* genome, 41,697 genes and 1768 ncRNAs were annotated (Table S3). Consistent with the genome assembly quality assessment, orthologs of 93.2% and 94.3% of the eukaryotic BUSCOs were identified in the *S. baicalensis* and *S. barbata* gene sets, respectively, suggesting the completeness of the genome annotation (Table S3). The gene-based synteny between *S. baicalensis* and *S. barbata* showed chromosome number variation and structural rearrangement (Figure 1B, Figure S3, Table S4). In addition, the alignment at the DNA sequence level also showed the large-scale structural variations between the *S. baicalensis* and *S. barbata* genomes (Figure S4).

### Chromosome rearrangements and expansion after speciation

Transposable elements (TEs) accounted for approximately 55.2% (208,004,279) and 53.5% (188,790,851) of the *S. baicalensis* and *S. barbata* genomes, respectively (Tables S5 and S6). Furthermore, 57.6% and 59.9% of these TEs were long terminal repeat (LTR) elements, respectively. We identified 1225 and 1654 full-length LTR elements, including *Gypsy* (342 and 310) and *Copia* (354 and 618) elements, in the *S. baicalensis* and *S. barbata* genomes, respectively (Table S7). However, there were differences in the insertion time of LTR elements, *i.e.*, the LTRs (1.41 million years ago; MYA) in *S. baicalensis* are more ancient than those in *S. barbata* (0.88 MYA), assuming a mutation rate of *μ* = 1.3 × 10^−8^ (per bp per year) (Figure S5, Table S7). The recent insertion and activation of LTRs might be key factors in the generation of chromosome rearrangements and expansion of *S. barbata* [30], [31]. The ribosomal RNAs (rRNAs) and simple sequence repeats (SSRs) were further annotated (Tables S8 and S9). A total of 142,951 and 147,705 SSRs were annotated in *S. baicalensis* and *S. barbata*, respectively. They will provide useful molecular markers for breeding and genetic diversity studies.

A genome-wide high-resolution Hi-C interaction analysis of *S. baicalensis* and *S. barbata* was performed to characterize the architectural features of folded eukaryotic chromatin, including interchromosomal interactions, compendium of chromosomal territories, and A/B compartments [32], [33], [34]. First, 159× and 173× Hi-C sequencing reads were uniquely mapped (49.6% and 59.0%) to the *S. baicalensis* and *S. barbata* reference genomes, respectively. Then, 84.8 and 113.1 million valid interaction pairs were obtained to construct the matrix of interactions among 100 kb binned genomic regions across all 9 *S. baicalensis* chromosomes and 13 *S. barbata* chromosomes. The whole-chromosome interactions of *S. baicalensis* indicated that chr5 and chr9 had a closer association than the other chromosome pairs. In *S. baicalensis*, the chromosome set including chr2, chr3, and chr8 showed enrichment and association with each other, and depletion with other interchromosomal sets, implying that these three chromosomes were mutually closer in space than the other chromosomes (Figure S6). In *S. barbata*, the chromosomal territories of chr4, chr5, and chr9 displayed mutual interactions and occupied an independent region in the nucleus (Figure S7).

As the secondary major structural unit of chromatin packing in *S. baicalensis* and *S. barbata*, the A/B compartments representing open and closed chromatin, respectively, were characterized according to an eigenvector analysis of the genome contact matrix. Similarly, more than half of the assembled *S. baicalensis* and *S. barbata* genomes (53.2% and 52.0%) were identified as the A compartment in the leaf tissue. As expected, the TE density in the A compartment was dramatically lower than that in the B compartment (*P* < 0.001), and the gene number per 100 kb was significantly higher in the A compartment (*P* < 0.001) (Figures S5 and S6), indicating a positive correlation between the A compartment and transcriptional activity or other functional measures [32], [34].

### Shared WGD events in Lamiaceae

Conserved sequences, including orthologs and paralogs, can be used to deduce evolutionary history based on whole-genome comparisons. Here, orthologous groups of amino acid sequences from 11 angiosperms were identified, yielding a total of 19,479 orthologous groups that covered 291,192 genes. Among these, 120,459 genes clustering in 6837 groups were conserved in all examined plants. Computational analysis of gene family evolution (CAFE) showed that 1180 and 1853 gene families were expanded in the *S. baicalensis* and *S. barbata* lineages, respectively, while 1599 and 1632 gene families were contracted, respectively (Figure S8, Table S10). Functional exploration of *Scutellaria*-specific genes indicated that domains related to secondary metabolite biosynthesis, such as transcription factors, cytochrome P450s, and O-methyltransferase were markedly enriched.

**Figure 2:**
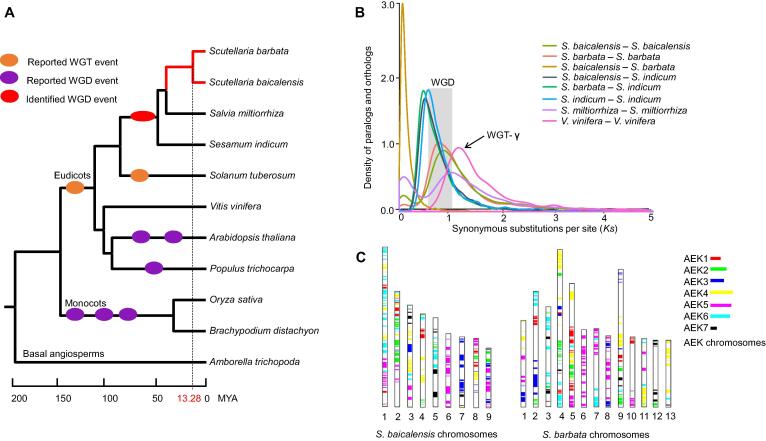
**Shared WGD events of Lamiaceae and Pedaliaceae** **A.** The phylogenetic tree based on the concatenated method using 235 single-copy orthologous genes from 11 angiosperms was constructed. The basal angiosperm *Amborella trichopoda* was chosen as the outgroup. The red branches represent two *Scutellaria* species, *S. baicalensis* and *S. barbata*. Speciation time was estimated based on the reported divergence time for *A. trichopoda*–*Vitis vinifera* (173–199 MYA) and *Populus trichocarpa*–*Arabidopsis thaliana* (98–117 MYA). The dashed line represents the divergence time between *S. baicalensis* and *S. barbata*. The orange ovals represent the reported WGT events. The purple and red ovals represent the reported WGD events and the newly identified WGD event in this study, respectively. The reported WGT/WGD events represent the WGT/WGD events identified in previous studies, including the WGT–γ event in core eudicots, WGT event in *Solabun tuberosum*, and WGD events in *Sesamum indicum*, *A. thaliana*, *P. trichocarpa*, *Oryze sativa*, and *Brachypodium distachyon*. **B.** Synonymous substitution rate (*Ks*) distributions of syntenic blocks for the paralogs and orthologs of *S. baicalensis*, *S. barbata*, *S. miltiorrhiza*, *S. indicum*, and *V. vinifera.* The gray box indicates the shared WGD event in *S. baicalensis*, *S. barbata*, *S. miltiorrhiza*, and *S. indicum.***C.** Comparison with AEK chromosomes. The syntenic AEK blocks are painted onto *S. baicalensis* and *S. barbata* chromosomes separately. MYA, million years ago; WGT, whole-genome triplication; WGD, whole-genome duplication; AEK, ancestral eudicot karyotype.

In addition, 235 single-copy genes were identified in all tested plants. They were used to construct a phylogenetic tree, which indicated that these two *Scutellaria* species were most closely related to *S. miltiorrhiza* with an estimated divergence time of 41.01 MYA. *S. baicalensis* and *S. barbata* were grouped into one branch, with an estimated divergence time of approximately 13.28 MYA (Figure 2A). The phylogenetic tree also supported the close relationship between Lamiaceae (*S. baicalensis*, *S. barbata*, and *S. miltiorrhiza*) and Pedaliaceae (*Sesamum indicum*) with a divergence time of approximately 49.90 MYA (Figure 2A) [35]. Previous research reported that the divergence time of *S. baicalensis* and *S. barbata* based on the *mat*K and *CHS* (chalcone synthase) genes was approximately 3.35 MYA [36]. However, a genome-wide analysis identified 8 and 3 *CHS* genes in *S. baicalensis* and *S. barbata*, respectively. The expansion and evolution of *CHS* negatively impacted the estimation of diversification history between these *Scutellaria* species.

Based on sequence homology, 17,265 orthologous gene pairs with synteny were identified between the *S. baicalensis* and *S. barbata* genomes. The distribution of synonymous substitution rates (*Ks*) peaked at approximately 0.16, representing the speciation time of *S. baicalensis* and *S. barbata* (Figure 2B, Table S11). The mean *Ks* values of orthologous gene pairs with synteny and the divergence time among *S. baicalensis*, *S. barbata*, *S. miltiorrhiza*, *S. indicum*, and *Vitis vinifera* [37] revealed the estimated synonymous substitutions per site per year as 1.30 × 10^−8^ for the test species (Table S11). In total, 7812, 7168, 6984, and 7711 paralogous gene pairs were identified, and the distribution of *Ks* values peaked at approximately 0.87, 0.86, 1.02, and 0.67 in *S. baicalensis*, *S. barbata*, *S. miltiorrhiza*, and *S. indicum*, respectively (Figure 2B, Table S11). Based on the phylogenetic analysis, the WGD event occurred prior to the divergence of *S. baicalensis*, *S. barbata*, *S. miltiorrhiza*, and *S. indicum*. The divergence time of the Lamiaceae and Pedaliaceae shared WGD event was determined to be approximately 46.24–60.71 MYA (Table S11). The distribution of the *Ks* values of paralogous genes showed that no WGD events have occurred since the divergence of *S. miltiorrhiza*, *S. baicalensis*, and *S. barbata*. Comparison of the *S. baicalensis* and *S. barbata* genomes with an ancestral eudicot karyotype (AEK) genome [38] and with the *V. vinifera* genome, also supported the structural rearrangement between the *S. baicalensis* and *S. barbata* genomes, and the shared WGD event after whole-genome triplication (WGT)-γ event of *V. vinifera* (Figure 2C, Figure S9). The genome syntenic analysis indicated two copies of syntenic blocks from Lamiaceae and Pedaliaceae species per corresponding *V. vinifera* block, which confirmed the recent WGD event before the divergence of *S. baicalensis*, *S. barbata*, and *S. indicum* (Figure S10).

### Organ-specific localization of bioactive compounds

Baicalein, scutellarein, norwogonin, wogonin, and their glycosides (baicalin, scutellarin, norwogonoside, and wogonoside) are the main bioactive compounds in *S. baicalensis* and *S. barbata*. We collected samples from the root, stem, leaf, and flower tissues of *S. baicalensis* and *S. barbata* to detect the accumulation of active compounds. Baicalein, norwogonin, wogonin, baicalin, norwogonoside, and wogonoside accumulated mainly in the roots of *S. baicalensis* and *S. barbata*, while scutellarin was distributed in the aerial parts (stem, leaf, and flower) of these species (Figure 1C, Figure S11, Table S12), providing a potential basis for the co-expression analysis of biosynthetic genes [23].

Transcriptome analysis of these four tissues from *S. baicalensis* and *S. barbata* included calculation of the fragments per kilobase of exon model per million reads mapped (FPKM) values of 39,121 and 47,200 genes, respectively. Among them, 31.5% (12,320) and 40.6% (19,153) of the transcripts were not expressed (FPKM < 1) in any of the tested tissues. Based on *k*-means clustering, all the expressed genes from *S. baicalensis* and *S. barbata* were classified into 48 clusters (Figures S12 and S13). The expression levels of 3421 genes from clusters 8, 20, 32, 33, 34, 39, and 47 in *S. baicalensis*, and 3675 genes from clusters 2, 4, 21, 25, 27, 31, and 40 in *S. barbata* were higher in the roots than in the other organs. The biosynthetic genes involved in the synthesis of *Scutellaria* specific flavones and glycosides, containing genes encoding chalcone synthase, chalcone isomerase, CYP450s, *O*-methyltransferase, glycosyltransferase, and glycosyl hydrolases, were enriched, with high expression in the roots of *S. baicalensis* and *S. barbata* (Tables S13 and S14).

### Conserved evolution of the chrysin and apigenin biosynthesis in *Scutellaria*

**Figure 3:**
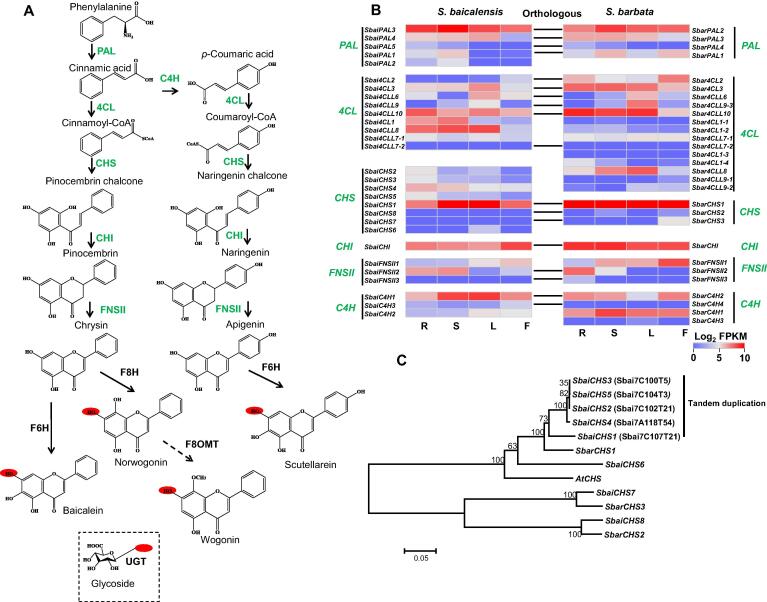
**Conserved flavonoid biosynthesis and species-specific gene expansion in *Scutellaria*** **A.** Genes related to the biosynthesis of flavones and their glycosides. The red ovals represent the hydroxyl groups that can be glucosylated by UGT. The dashed box means the glycoside. **B.** The expression profile and orthologous gene pairs of flavone biosynthetic genes in *S. baicalensis* and *S. barbata*. **C.** Tandem duplication and phylogenetic analysis of *CHS* genes. The phylogenetic tree was constructed based on maximum likelihood method. PAL, phenylalanine ammonia lyase; C4H, cinnamate 4-hydroxylase; 4CL, 4-CoA ligase; CHS, chalcone synthase; CHI, chalcone isomerase; FNSII, flavone synthase II; F6H, flavone 6-hydroxylase; F8H, flavone 8-hydroxylase; UGT, UDP-glycosyltransferase; F8OMT, flavone 8-*O*-methyltransferase.

The main active compounds in the medicinal plants *S. baicalensis* and *S. barbata* are flavonoids. The chrysin biosynthetic genes in *S. baicalensis* encoding 4-CoA ligase (4CL), CHS, chalcone isomerase (CHI), and flavone synthase (FNSII) have been cloned and functionally identified [14]. However, the gene locations, gene numbers, and evolution of this pathway in the *S. baicalensis* and *S. barbata* genomes remain unclear. Here, we identified the same number of chrysin and apigenin biosynthetic genes in the *S. baicalensis* and *S. barbata* genomes and determined the expression levels of these genes, including phenylalanine ammonia lyase (*PAL*, 5 and 4), cinnamate 4-hydroxylase (*C4H*, 3 and 4), *4CL* (9 and 14), *CHS* (8 and 3), *CHI* (1 and 1), and *FNSII* (3 and 3), in different tissues (Figure 3A and B, Tables S15 and S16). Eighteen orthologous gene pairs were found between the *S. baicalensis* and *S. barbata* genomes, and the *Ka*/*Ks* value (average 0.13) indicated purifying selection on flavone biosynthesis during evolution [39] (Figure 3B, Table S17). The *PAL* and *CHS* gene numbers in *S. baicalensis* were expanded compared to those in *S. barbata*. Conversely, a duplication event of *4CL* genes in *S. barbata* was found, suggesting that expansion via tandem duplication might have occurred after the separation of these *Scutellaria* species. The *Ks* values of 18 orthologous gene pairs of *S. baicalensis* and *S. barbata* in the chrysin and apigenin biosynthetic pathways indicated that the specific expansion of the *SbaiPAL* (*SbaiPAL1* and *SbaiPAL2*), *SbaiCHS* (*SbaiCHS2*, *SbaiCHS3*, *SbaiCHS4*, and *SbaiCHS5*), and *Sbar4CL* (*Sbar4CL1-1*, *Sbar4CL1-2*, *Sbar4CL1-3*, *Sbar4CL1-4*, *Sbar4CLL9-2*, and *Sbar4CLL9-3*) genes had occurred via tandem duplication, after the speciation of *S. baicalensis* and *S. barbata* (Figure 3C, Figure S14, Table S17).

*Sbai4CLL7* and *SbaiCHS1* are reportedly related to the biosynthesis of specific 4′-deoxyflavones with cinnamic acid as a substrate in *S. baicalensis* [14]. Compared to *S. miltiorrhiza*, the *4CLL7* genes from the *Scutellaria* genus showed gene expansion, and the gene duplication of *Sbai4CLL7-1* and *Sbai4CLL7-2* occurred before the speciation of *S. baicalensis* and *S. barbata* (Figure S13). *Sbai4CLL7-1* and *Sbar4CLL7-1* were not expressed in the tested transcriptomes, and the duplication of the *Scutellaria*-specific *4CLL7-2* allowed the evolution of substrate preferences for the catalysis of cinnamic acid. The initial step and central hub for flavone biosynthesis is the catalysis of CHS. Hence, the expression of *CHS* is required for the production of flavonoids, isoflavonoids, and other metabolites in plants [40]. Here, we also detected the highest expression levels of *SbaiCHS1* and *SbarCHS1* in all the tested samples. However, a recent expansion of *CHS* genes has occurred in *S. baicalensis*, and 4 additional paralogs of *SbaiCHS1* (*Sbai7C107T21*) were observed in chr7. Duplications of the *SbaiCHS2*, *SbaiCHS3*, *SbaiCHS4,* and *SbaiCHS5* genes occurred after the speciation of *S. baicalensis* and *S. barbata* (Figure 3C). The nucleotide and amino acid sequences of *SbaiCHS2* and *SbaiCHS3* were identical, but *SbaiCHS5* contained a variant K316 deletion. The divergence of *SbaiCHS1* and *SbarCHS1* occurred before the separation of *S. miltiorrhiza* and the *Scutellaria* species, suggesting a conserved function of CHS in flavone biosynthesis. In addition, the tandemly duplicated *SbaiCHS2-5* genes were more highly expressed in the root of *S. baicalensis* than in other tissues (Figure 3B), suggesting that their species-specific evolution might be related to the biosynthesis of flavones and their glycosides, which are enriched in root.

C4H is responsible for the biosynthesis of coumaroyl-CoA, which might be the restrictive precursor of the 4′-hydroxyl group involved in scutellarein biosynthesis. *SbaiC4H1* and *SbarC4H1* were highly expressed in the stems, leaves, and flowers of *S. baicalensis* and *S. barbata* (Figure 3B, Figure S14). The high expression levels of these genes were positively correlated with the distribution of scutellarin, which is biosynthesized in the aerial parts of *S. baicalensis* and *S. barbata* (Figure 1C).

SbaiFNSII2 has been reported to catalyze the formation of chrysin in *S. baicalensis* [14], and the coding gene was highly expressed in the root and stem. Its ortholog, *SbarFNSII2*, was also highly expressed in the root of *S. barbata*. A genome colinearity analysis identified 566 orthologous gene pairs covering a region approximately 6 Mb in length across chr3 of *S. baicalensis* and chr13 of *S. barbata*, including the tandem duplication of *SbaiFNSII1*–*SbaiFNSII2* and *SbarFNSII1*–*SbarFNSII2*. This duplication occurred before the speciation of *S. baicalensis* and *S. barbata* (Figure S14). The majority of the *FNSII* region (approximately 85%) in *S. baicalensis* and *S. barbata* was assigned to the A compartment, indicating high transcriptional activity. The genome synteny of the *FNSII* region between *S. baicalensis* and *S. barbata* suggested the conserved evolution of flavone synthase.

### Functional divergence of flavone hydroxylase genes between ***S. baicalensis*** and ***S. barbata***

**Figure 4:**
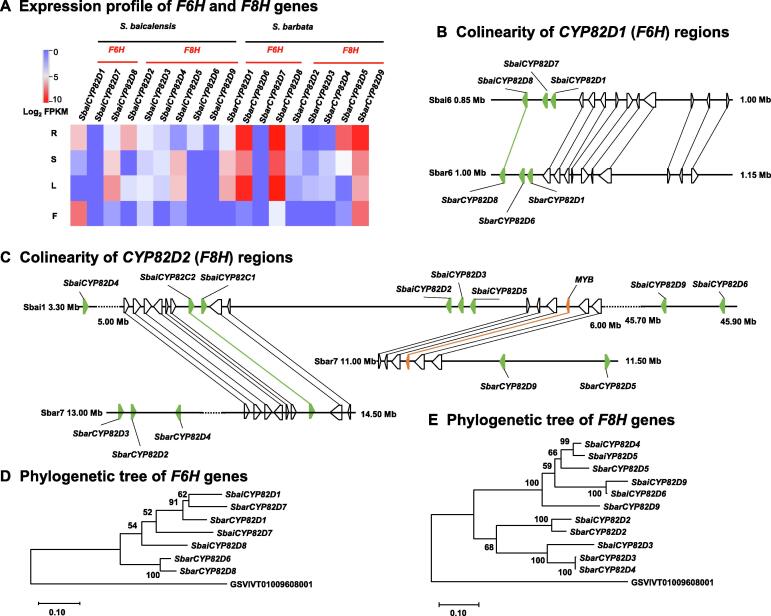
**Tandem repeat of flavone hydroxylase genes revealed the divergent evolution** **A.** Identification and expression of *CYP82D* subfamily genes, including *F6H* and *F8H*. **B.** Colinearity of *CYP82D1* (*F6H*) regions between *S. baicalensis* and *S. barbata*. **C.** Colinearity of *CYP82D2* (*F8H*) regions between *S. baicalensis* and *S. barbata*. The green triangles represent *CYP450* genes. The orange triangle represents *MYB* transcription factor gene. The black triangles indicate neighboring genes of the selected *CYP450* genes. **D.** Phylogenetic tree of *F6H* genes. **E.** Phylogenetic tree of *F8H* genes. Grape *CYP82D* (GSVIVT01009608001, http://plants.ensembl.org/Vitis_vinifera) was chosen as the outgroup to generate both trees. MYB, my elob lastosis.

CYP450 superfamily members, such as C4H (CYP73A family), FNSII (CYP93B family), flavone 6-hydroxylase (F6H, CYP82D family), and flavone 8-hydroxylase (F8H, CYP82D family), perform key modifications in flavone biosynthesis. SbaiCYP82D1 has been reported to have 6-hydroxylase activity on chrysin and apigenin to produce baicalein and scutellarein, respectively, and SbaiCYP82D2 can catalyze chrysin to norwogonin in *S. baicalensis* [15] (Figure S15). Here, we identified 418 and 398 *CYP450* gene members, and 17 and 24 physical clusters of *CYP450s* (5 gene clusters per 500 kb) in the *S. baicalensis* and *S. barbata* genomes, respectively (Figures S16 and S17), suggesting a high frequency of *CYP* gene tandem duplication. Among them, 18 *CYP82D* members containing *SbaiCYP82D1-9* and *SbarCYP82D1-9* were identified in the *S. baicalensis* and *S. barbata* genomes. These genes might be responsible for the hydroxylation of chrysin and apigenin (Table S18). Consistent with a previous report, high expression of *SbaiCYP82D1* and *SbaiCYP82D2* in the root of *S. baicalensis* was detected, in accordance with the accumulation of baicalein, wogonin, and their glycosides (Figure 4A). However, *SbarCYP82D1* showed relatively high expression in stems and leaves, and *SbarCYP82D2* showed extremely low expression in all tissues of *S. barbata*, in contrast to the distributions of active flavones, suggesting a potential functional divergence of hydroxylation between *S. baicalensis* and *S. barbata*.

Three-gene tandem duplications of *SbaiCYP82D1*–*SbaiCYP82D7*–*SbaiCYP82D8* and *SbarCYP82D1*–*SbarCYP82D6*–*SbarCYP82D8* (physical distance  < 30 kb) on chr6 of *S. baicalensis* and *S. barbata* were identified (Figure 4B). According to the 150 kb colinearity analysis, 11 orthologous gene pairs, including *CYP82D8* from *S. baicalensis* and *S. barbata*, presented conserved evolution. The phylogenetic analysis and *Ks* values of orthologous gene pairs indicated that the duplication of *SbarCYP82D8* and *SbarCYP82D6* occurred after the speciation of *S. barbata* (Table S19). However, duplication of *SbaiCYP82D8* and *SbaiCYP82D7* occurred before the divergence of *S. baicalensis* and *S. barbata* (Figure 4D, Figure S18). This contradiction and evolutionary divergence support the following proposed hypothesis, which features three duplications. The first duplication of *CYP82D8* produced the new *CYP82D1*, and the duplication event occurred around WGD event. The second duplication of *CYP82D8* generated the new *CYP82D7*, similar to the tandem duplication of *SbaiCYP82D8*–*SbaiCYP82D7*–*SbaiCYP82D1* in *S. baicalensis*. After speciation, the third duplication event of *SbarCYP82D8* uniquely occurred in the *S. barbata* genome and produced *SbarCYP82D6*; a recent gene transfer of *SbarCYP82D7* via transposon from chr6 to chr3 in *S. barbata* was predicted. An adjacent intact LTR/*Gypsy* in *SbarCYP82D7* was identified, and its insertion time was estimated to be approximately 3.5 MYA. Given the evolution and high expression of *SbarCYP82D6* and *SbarCYP82D8*, we speculate that these two genes might be responsible for the F6H function in chrysin and apigenin synthesis *in vivo* in *S. barbata*.

The chromosome location of F8H functional members showed that *SbaiCYP82D2*, *SbaiCYP82D3*, *SbaiCYP82D4*, *SbaiCYP82D5*, *SbaiCYP82D6*, and *SbaiCYP82D9* were distributed on chr1 of *S. baicalensis*, with *SbarCYP82D2*, *SbarCYP82D3*, *SbarCYP82D4*, *SbarCYP82D5*, and *SbarCYP82D9* located on chr7 of *S. barbata*. The structural rearrangement of large segments between chr1 of *S. baicalensis* and chr7 of *S. barbata* was found (Figure 4C, Figure S4). In addition, tandem duplications containing three *CYP* genes (*SbaiCYP82D2*–*SbaiCYP82D3*–*SbaiCYP82D5* and *SbarCYP82D3*–*SbarCYP82D2*–*SbarCYP82D4*) were identified (Figure 4C). The orthologous gene pairs *SbaiCYP82D2*–*SbarCYP82D2* and *SbaiCYP82D3*–*SbarCYP82D3* presented high identity values of 90.11% and 83.72%, respectively. The duplications of *SbarCYP82D3*–*SbarCYP82D4*, *SbaiCYP82D4*–*SbaiCYP82D5*, and *SbaiCYP82D6*–*SbaiCYP82D9* occurred after the speciation of *S. baicalensis* and *S. barbata* (Table S19). However, the expression leveles of *SbarCYP82D2*, *SbarCYP82D3*, and *SbarCYP82D4* were low in *S. barbata*, indicating functional divergence following species-specific duplication events. In contrast, *SbarCYP82D5* and *SbarCYP82D9* were highly expressed in the root of *S. barbata*, suggesting a potential F8H function in the biosynthesis of norwogonin.

## Conclusion

We reported two chromosome-level genomes of the medicinal plants *S. baicalensis* and *S. barbata* through the combination of second-generation sequencing (Illumina platform), third-generation sequencing (PacBio and Oxford Nanopore platforms), and Hi-C technologies. This study confirmed and traced the divergence time of *S. baicalensis* and *S. barbata*, which occurred at 13.28 MYA, far earlier than previously reported [36]. Comparative genomic analysis revealed similar TE proportions in the *S. baicalensis* and *S. barbata* genomes, while the recent LTR insertion in *S. barbata* might be an important factor resulting in chromosomal rearrangement and expansion. A WGD event (approximately 42.64–60.71 MYA) shared among *S. baicalensis*, *S. barbata*, *S. miltiorrhiza*, and *S. indicum*. The tandem duplication of paralogs after the speciation of *S. baicalensis* and *S. barbata* might be the most important contributor to the divergent evolution of flavonoid biosynthetic gene families, such as *PAL*, *4CL*, *CHS*, *F6H*, and *F8H*. A determination of the distribution of flavone content and transcriptome analysis supported the functional divergence of flavonoid biosynthetic genes between *S. baicalensis* and *S. barbata*. The two high-quality genomes reported in this study will enrich genome research in the Lamiaceae and provide important insights for studies of breeding, evolution, chemodiversity, and genome editing.

## Materials and methods

### Plant materials

*S. baicalensis* and *S. barbata* plants were cultivated in the experimental field (40 °N and 116 °E) of the Institute of Medicinal Plant Development, Chinese Academy of Medical Sciences & Peking Union Medical College, Beijing, China. Four independent tissues (root, stem, leaf, and flower) from *S. baicalensis* and *S. barbata* were collected in three replicates. These tissues were used separately for the measurement of active compounds and RNA-seq. High-quality DNA extracted from young leaves was used to construct libraries for Illumina, ONT, and Sequel sequencing.

### Long-read sequencing and assemblies

The high-molecular-weight (HMW) genomic DNA of *S. baicalensis* and *S. barbata* was extracted as described for megabase-sized DNA preparation [41]. HMW genomic DNA fragments (>20 kb) were selected using BluePippin. Long-read libraries were constructed following the protocols for the ONT (https://nanoporetech.com/) and PacBio Sequel platforms (https://www.pacb.com/). The ONT reads of *S. baicalensis* were generated using the ONT GridION X5 platform, and the library of *S. barbata* was sequenced using the Sequel platform. The raw ONT and SMRT reads were filtered via MinKNOW and SMRT Link, respectively. First, Canu (v1.7) was used to correct and trim the long reads from the ONT and Sequel platforms with the default parameters [42]. The corrected and trimmed ONT and SMRT reads were assembled using SMARTdenovo (https://github.com/ruanjue/smartdenovo). Finally, Illumina short reads were used to polish the assembled contigs three times using Pilon (v1.22). The quality of the genome assemblies was estimated by a search using BUSCO (v2.0) [43] and by mapping Illumina reads from the DNA and RNA libraries to the assembled genomes.

### Chromosome construction using Hi-C

Young leaves from *S. baicalensis* and *S. barbata* were fixed and crosslinked, and Hi-C libraries were constructed and sequenced using Illumina as previously described [32], [33]. The short reads were mapped to the assembled genome using BWA [44], and the valid interaction pairs were selected using HiC-Pro [45]. The draft assemblies of *S. baicalensis* and *S. barbata* were anchored to chromosomes (2*n* = 18 and 2*n* = 26, respectively) using LACHESIS with the following parameters: cluster min re sites = 62, cluster max link density = 2, cluster noninformative ratio = 2, order min n res in turn = 53, order min n res in shreds = 52 [29].

### Genome annotation

The RepeatModeler (v1.0.9) package, including RECON and RepeatScout, was used to identify and classify the repeat elements of the *S. baicalensis* and *S. barbata* genomes. The repeat elements were then masked by RepeatMasker (v4.0.6). The long terminal repeat retrotransposons (LTR-RTs) in *S. baicalensis* and *S. barbata* were identified using LTR_Finder (v1.0.6) and LTR_retriever. Twenty-four samples from a total of eight different *S. baicalensis* and *S. barbata* tissues (root, stem, leave, and flower) were subjected to RNA-seq using the Illumina NovaSeq platform. The clean reads from *S. baicalensis* and *S. barbata* were *de novo* assembled using Trinity (v2.2.0), and the coding regions in the assembled transcripts were predicted using TransDecoder (v2.1.0). The gene annotation of the masked *S. baicalensis* and *S. barbata* genomes was *ab initio* predicted using the MAKER (v2.31.9) pipeline, integrating the assembled transcripts and protein sequences from *S. baicalensis*, *S. barbata*, and *Arabidopsis thaliana* [46]. Noncoding RNAs and miRNAs were annotated by alignment to the Rfam and miRNA databases using INFERNAL (v1.1.2) and BLASTN, respectively. RNA-seq reads from different *S. baicalensis* and *S. barbata* tissues were mapped to the masked genome using HISAT2 (v2.0.5), and the different expression levels of the annotated genes were calculated using Cufflinks (v2.2.1) [47].

### Genome evolution analysis

The full amino acid sequences of *S. baicalensis*, *S. barbata*, and nine other angiosperms were aligned to orthologous groups using OrthoFinder [48]. The basal angiosperm *Amborella trichopoda* was chosen as the outgroup. Single-copy genes were used to construct a phylogenetic tree using the RAxML package with PROTGAMMAJTT model and 1000 replicates (v8.1.13). The divergence time among 11 plants was predicted using r8s program based on the estimated divergence time *A. trichopoda*–*V. vinifera* (173–199 MYA) and *Populus trichocarpa*–*A. thaliana* (98–117 MYA). According to the phylogenetic analysis and divergence time, expansion and contraction of the gene families were identified using CAFE (v3.1) [49]. The paralogous and orthologous gene pairs from *S. baicalensis*, *S. barbata*, and *S. miltiorrhiza* were identified, and the *Ka*, *Ks*, and *Ka/Ks* values of *S. baicalensis*–*S. baicalensis*, *S. barbata*–*S. barbata*, *S. miltiorrhiza*–*S. miltiorrhiza*, *S. baicalensis*–*S. miltiorrhiza*, *S. baicalensis*–*S. barbata*, and *S. barbata*–*S. miltiorrhiza* were calculated using the SynMap2 and DAGchainer method of the CoGE Comparative Genomics Platform (https://genomevolution.org/coge/). The detection of synteny and colinearity among *S. baicalensis*, *S. barbata*, and *S. miltiorrhiza* was performed using MCscan X(v1.1) [50]. The WGT–γ event in core eudicots, WGT event in *S. tuberosum*, and WGD events in *S. indicum*, *A. thaliana*, *P. trichocarpa*, *Oryze sativa*, and *Brachypodium distachyon* [51] were marked in our phylogenetic tree.

### Identification of gene families related to flavone biosynthesis

The protein sequences of the *PAL*, *4CL*, *C4H*, *CHS*, *CHI*, and *FNSII* gene family members in *A. thaliana* were downloaded from the TAIR database, and those for *F6H* and *F8H* in *S. baicalensis* were obtained from a previous study [15]. These sequences were searched against the *S. baicalensis* and *S. barbata* protein sequences using BLASTP with an *E* value cutoff of 1E–10. The conserved domains of the protein sequences of candidate genes were further searched in the Pfam database using hidden Markov models [52]. Full-length protein sequences were used to construct phylogenetic trees using the maximum likelihood method with the Jones-Taylor-Thornton model and 1000 bootstrap replicates [53]. A detailed description of some materials and methods used is provided in the supplementary methods and results.

## Data availability

The raw sequence data reported in this paper have been deposited in the Genome Sequence Archive [54] in the National Genomics Data Center, Beijing Institute of Genomics, Chinese Academy of Sciences / China National Center for Bioinformation (GSA: CRA001730) that are publicly accessible at http://bigd.big.ac.cn/gsa. The assembled genomes and gene structures were submitted to CoGe (https://genomevolution.org/coge/) with ID 54175 for *S. baicalensis* and ID 54176 for *S. barbata*. The assembled genomes and gene structures have also been deposited in the Genome Warehouse (GWHAOTO00000000 for *S. baicalensis* and GWHAOTP00000000 for *S. barbata*), which are publicly accessible at https://bigd.big.ac.cn/gwh.

## CRediT author statement

**Zhichao Xu:** Formal analysis, Writing - original draft, Writing - review & editing, Visualization, Project administration, Funding acquisition. **Ranran Gao:** Formal analysis, Writing - original draft, Writing - review & editing, Visualization. **Xiangdong Pu:** Formal analysis. **Rong Xu:** Resources. **Jiyong Wang:** Resources. **Sihao Zheng:** Resources. **Yan Zeng:** Resources. **Jun Chen:** Resources. **Chunnian He:** Validation. **Jingyuan Song:** Supervision, Project administration, Funding acquisition. All authors read and approved the final manuscript.

## Competing interests

The authors have declared no competing interests.

## Supplementary material

Supplementary File S1 Supplementary methods and results.

Supplementary Figure S1 **Genome size estimation. A.** Flow cytometry analysis using *Salvia miltiorrhiza* data as internal standards. The number of nuclei counted in tested samples is indicated on the Y axis. The relative fluorescence of FL2-A (585/40 nm) is displayed on the X axis. **B.** The 21 *k*-mer distribution from Illumina short reads of *S. baicalensis* and *S. barbata*.

Supplementary Figure S2 **Hi-C intrachromosomal contact map**. The red diagonal line indicates a high number of intrachromosomal contacts. **A.** Hi-C heatmap of *S. baicalensis*. Number of links at the 100-kb resolution is indicated. **B.** Hi-C heatmap of *S. barbata*. Hi-C, high-through chromosome conformation capture.

Supplementary Figure S3 **Genome synteny analysis.** The genome synteny results were analyzed using MCScanX between *S. baicalensis* and *S. barbata* (**A**), *S. baicalensis* and *S. indicum* (**B**), *S. barbata* and *S. indicum* (**C**), respectively.

Supplementary Figure S4 **Alignment of large-scale DNA sequences**. The dot plot presented the genome alignment between *S. baicalensis* and *S. barbata* using MUMmer with the minimum mapping length of 100 kb. The red dots represent forward matches, and the blue dots represent reverse matches.

Supplementary Figure S5 **Insertion time distribution of intact LTR-RTs. A.** Difference of LTR-RT distribution between *S. baicalensis* and *S. barbata*. **B.** Difference of insertion time of LTR-RTs between *S. baicalensis* and *S. barbata*. LTR-RT, long terminal repeat retrotransposon.

Supplementary Figure S6 **Genome-wide chromatin packing analysis in S. baicalensis. A.** The intrachromosomal interactions revealing the A compartments (red boxes) and B (blue boxes) compartments of *S. baicalensis*. **B.** The ratio of TE and gene numbers between the A and B compartments. **C.** The interchromosomal interactions of *S. baicalensis*. The heatmap based on Log2(Obs/Exp) indicates that the chromatin interaction frequencies are transformed into normalized values. TE, transposable element; Obs, observed; Exp, expected.

Supplementary Figure S7 **Genome-wide chromatin packing analysis in *S. barbata*. A.** The intrachromosomal interactions revealing the A (red boxes) and B (blue boxes) compartments of *S. barbata*. **B.** The ratio of TE and gene numbers between the A and B compartments. **C.** The interchromosomal interactions of *S. barbata*. The heatmap based on Log2(Obs/Exp) indicates that the chromatin interaction frequencies are transformed into normalized observed/expected values. TE, transposable element; Obs, observed; Exp, expected.

Supplementary Figure S8 **Gene family expansion and contraction.** The numbers of each branch represent the gene family expansion (in red) and contraction (in green). The number of expansion, remain, and contraction events of 20 nodes is also listed in Table S10.

Supplementary Figure S9 **Synteny analysis against *V. vinifera* genome.** The *V. vinifera* genome was painted into *S. baicalensis* and *S. barbata* genome, respectively, based on the gene collinearity using MCScanX.

Supplementary Figure S10 **Genome synteny analysis among related species.** Dot plots presented the gene synteny between *V. vinifera* and *S. indicum* (**A**), *V. vinifera* and *S. baicalensis* (**B**), *V. vinifera* and *S. barbata* (**C**), respectively. The red circles highlighted the duplication events after WGT-γ event. Dot plots of paralogs in *S. indicum* (**D**), *S. baicalensis* (**E**), and *S. barbata* (**F**) to show the potential duplication events.

Supplementary Figure S11 **UPLC detection of flavonoid contents.** UPLC detection of flavonoids (280 nm) in different tissues of *S. baicalensis* and *S. barbata*, including baicalein, scutellarein, wogonin, and their glycosides (baicalin, scutellarin, and wogonoside). The compound information, including detailed retention time and spectrum data, is listed in Table S12. **A**. Flavonoid contents of *S. baicalensis*. **B**. Flavonoid contents of *S. barbata*.

Supplementary Figure S12 **Gene expression clusters in *S. baicalensis*.** All expressed genes were classified into 48 clusters based on *k*-means in different *S. baicalensis* tissues, namely, root, stem, leaf, and flower tissues.

Supplementary Figure S13 **Gene expression clusters in *S. barbata*.** All expressed genes were classified into 48 clusters based on *k*-means in different *S. barbata* tissues, namely, root, stem, leaf, and flower tissues.

Supplementary Figure S14 **Phylogenetic analysis of *PAL*, *C4H*, *4CL*, and *FNSII*.** The phylogenetic trees for *PAL* (**A**), *C4H* (**B**), *4CL* (**C**), and *FNSII* (**D**) genes were constructed using the maximum likelihood method with bootstrap of 1000 times.

Supplementary Figure S15 **Potential downstream biosynthetic pathway.** The biosynthesis of baicalein, scutellarein, wogonin, and their glycosides (baicalin, scutellarin, and wogonoside). **A**. Chrysin as substrate. F6H catalyzes chrysin to produce baicalein. F8H transforms chrysin to norwogonin, and F8OMT further catalyzes norwogonin to wogonin. Then, UBGAT perform the transfer of glucuronic acid to the 7-OH of baicalein, norwogonin, and wogonin. **B**. Apigenin as substrate. F6H transforms apigenin to scutellarein, then UBGAT catalyzes the transfer of glucuronic acid to the 7-OH of scutellarein to produce scutellarin. F6H, flavone 6-hydroxylase; F8H, flavone 8-hydroxylase; F8OMT, flavone 8-*O*-methyltransferases; UGAT, UDP- glucuronosyltransferase.

Supplementary Figure S16 **Physical clusters of *CYP450* genes in *S. baicalensis*.** Regions with > 5 gene clusters per 500 kb are marked with red stars.

Supplementary Figure S17 **Physical clusters of *CYP450* genes in *S. barbata***. Regions with > 5 gene clusters per 500 kb are marked with red stars.

Supplementary Figure S18 **Phylogenetic analysis of *CYP82D*, *CYP93B*, and *CYP73A* members**. The phylogenetic trees of *CYP82D*, *CYP93B*, and *CYP73A* members from *S. baicalensis* and *S. barbata* were constructed using the maximum likelihood method with bootstrap of 1000 times.

Supplementary Table S1 **Statistics of sequencing****data**.

Supplementary Table S2 **Statistics of *Scutellaria* genome****assembly**.

Supplementary Table S3 **Statistics of genome****annotations.**

Supplementary Table S4 **Genome synteny between *S. baicalensis* and*****S. barbata*.**

Supplementary Table S5 **Annotation of *S. baicalensis*****TEs.**

Supplementary Table S6 **Annotation of *S. barbata*****TEs.**

Supplementary Table S7 **Summary of intact LTR****retrotransposons.**

Supplementary Table S8 **Annotation of *S. baicalensis* and *S. barbata* rRNA****genes.**

Supplementary Table S9 **Identification of****SSRs.**

Supplementary Table S10 **Gene family expansion and****contraction.**

Supplementary Table S11 **The *Ks* value and divergence time of paralogous or orthologous gene****pairs.**

Supplementary Table S12 **Compound information of****UPLC detection.**

Supplementary Table S13 **Pfam annotation of genes with high expression in the root of*****S. baicalensis*.**

Supplementary Table S14 **Pfam annotation of genes with high expression in the root of*****S. barbata*.**

Supplementary Table S15 **Expression of chrysin and apigenin biosynthetic genes in*****S. baicalensis*.**

Supplementary Table S16 **Expression of chrysin and apigenin biosynthetic genes in*****S. barbata*.**

Supplementary Table S17 ***Ka* and *Ks* analysis of chrysin and apigenin biosynthetic****genes.**

Supplementary Table S18 **Expression of *CYP82D*****members.**

Supplementary Table S19 ***Ks* values of gene pairs related to flavone****biosynthesis.**

## References

[1] Winkel-Shirley B. (2001). Flavonoid biosynthesis. A colorful model for genetics, biochemistry, cell biology, and biotechnology. Plant Physiol.

[2] Winkel-Shirley B. (2002). Biosynthesis of flavonoids and effects of stress. Curr Opin Plant Biol.

[3] Grotewold E. (2006). The genetics and biochemistry of floral pigments. Annu Rev Plant Biol.

[4] Shang X.F., He X.R., He X.Y., Li M.X., Zhang R.X., Fan P.C. (2010). The genus *Scutellaria* an ethnopharmacological and phytochemical review. J Ethnopharmacol.

[5] Grzegorczyk-Karolak I., Wiktorek-Smagur A., Hnatuszko-Konka K. (2018). An untapped resource in the spotlight of medicinal biotechnology: the genus *Scutellaria*. Curr Pharm Biotechnol.

[6] Chinese Pharmacopoeia Commission (2015). Pharmacopoeia of the People’s Republic of China.

[7] Zhang Z., Lian X.Y., Li S., Stringer J.L. (2009). Characterization of chemical ingredients and anticonvulsant activity of American skullcap (*Scutellaria lateriflora*). Phytomedicine.

[8] Qiao X., Li R., Song W., Miao W.J., Liu J., Chen H.B. (2016). A targeted strategy to analyze untargeted mass spectral data: rapid chemical profiling of *Scutellaria baicalensis* using ultra-high performance liquid chromatography coupled with hybrid quadrupole orbitrap mass spectrometry and key ion filtering. J Chromatogr A.

[9] Yan B.F., Xu W.J., Su S.L., Zhu S.Q., Zhu Z.H., Zeng H.T. (2017). Comparative analysis of 15 chemical constituents in *Scutellaria baicalensis* stem-leaf from different regions in China by ultra-high performance liquid chromatography with triple quadrupole tandem mass spectrometry. J Sep Sci.

[10] Zhao Q., Chen X.Y., Martin C. (2016). *Scutellaria baicalensis*, the golden herb from the garden of Chinese medicinal plants. Sci Bull.

[11] Dai J.Y., Liang K., Zhao S., Jia W.T., Liu Y., Wu H.K. (2018). Chemoproteomics reveals baicalin activates hepatic CPT1 to ameliorate diet-induced obesity and hepatic steatosis. Proc Natl Acad Sci U S A.

[12] Guo H.X., Liu D.H., Ma Y., Liu J.F., Wang Y., Du Z.Y. (2009). Long-term baicalin administration ameliorates metabolic disorders and hepatic steatosis in rats given a high-fat diet. Acta Pharmacol Sin.

[13] Chen S.L., Song J.Y., Sun C., Xu J., Zhu Y.J., Verpoorte R. (2015). Herbal genomics: examining the biology of traditional medicines. Science.

[14] Zhao Q., Zhang Y., Wang G., Hill L., Weng J.K., Chen X.Y. (2016). A specialized flavone biosynthetic pathway has evolved in the medicinal plant, *Scutellaria baicalensis*. Sci Adv.

[15] Zhao Q., Cui M.Y., Levsh O., Yang D., Liu J., Li J. (2018). Two CYP82D enzymes function as flavone hydroxylases in the biosynthesis of root-specific 4'-deoxyflavones in *Scutellaria baicalensis*. Mol Plant.

[16] Liu X.N., Cheng J., Zhang G.H., Ding W.T., Duan L.J., Yang J. (2018). Engineering yeast for the production of breviscapine by genomic analysis and synthetic biology approaches. Nat Commun.

[17] Li J.H., Tian C.F., Xia Y.H., Mutanda I., Wang K.B., Wang Y. (2019). Production of plant-specific flavones baicalein and scutellarein in an engineered *E. coli* from available phenylalanine and tyrosine. Metab Eng.

[18] Xu Z.C., Song J.Y. (2017). The 2-oxoglutarate-dependent dioxygenase superfamily participates in tanshinone production in *Salvia miltiorrhiza*. J Exp Bot.

[19] Cao W.Z., Wang Y., Shi M., Hao X.L., Zhao W.W., Wang Y. (2018). Transcription factor SmWRKY1 positively promotes the biosynthesis of tanshinones in *Salvia miltiorrhiza*. Front Plant Sci.

[20] Huang Q., Sun M.H., Yuan T.P., Wang Y., Shi M., Lu S.J. (2019). The AP2/ERF transcription factor SmERF1L1 regulates the biosynthesis of tanshinones and phenolic acids in *Salvia miltiorrhiza*. Food Chem.

[21] Sun M.H., Shi M., Wang Y., Huang Q., Yuan T.P., Wang Q. (2019). The biosynthesis of phenolic acids is positively regulated by the JA-responsive transcription factor ERF115 in *Salvia miltiorrhiza*. J Exp Bot.

[22] Xu H.B., Song J.Y., Luo H.M., Zhang Y.J., Li Q.S., Zhu Y.J. (2016). Analysis of the genome sequence of the medicinal plant *Salvia miltiorrhiza*. Mol Plant.

[23] Xu Z.C., Peters R.J., Weirather J., Luo H.M., Liao B.S., Zhang X. (2015). Full-length transcriptome sequences and splice variants obtained by a combination of sequencing platforms applied to different root tissues of *Salvia miltiorrhiza* and tanshinone biosynthesis. Plant J.

[24] Xin T.Y., Zhang Y., Pu X.D., Gao R.R., Xu Z.C., Song J.Y. (2019). Trends in herbgenomics. Sci China Life Sci.

[25] Xu Z.C., Xin T.Y., Bartels D., Li Y., Gu W., Yao H. (2018). Genome analysis of the ancient tracheophyte *Selaginella tamariscina* reveals evolutionary features relevant to the acquisition of desiccation tolerance. Mol Plant.

[26] Schmidt M.H., Vogel A., Denton A.K., Istace B., Wormit A., van de Geest H. (2017). *De novo* assembly of a new *Solanum pennellii* accession using nanopore sequencing. Plant Cell.

[27] Guo L., Winzer T., Yang X.F., Li Y., Ning Z.M., He Z.S. (2018). The opium poppy genome and morphinan production. Science.

[28] Vining K.J., Johnson S.R., Ahkami A., Lange I., Parrish A.N., Trapp S.C. (2017). Draft genome sequence of *Mentha longifolia* and development of resources for mint cultivar improvement. Mol Plant.

[29] Burton J.N., Adey A., Patwardhan R.P., Qiu R., Kitzman J.O., Shendure J. (2013). Chromosome-scale scaffolding of *de novo* genome assemblies based on chromatin interactions. Nat Biotechnol.

[30] VanBuren R., Wai C.M., Ou S., Pardo J., Bryant D., Jiang N. (2018). Extreme haplotype variation in the desiccation-tolerant clubmoss *Selaginella lepidophylla*. Nat Commun.

[31] Bennetzen J.L., Wang H. (2014). The contributions of transposable elements to the structure, function, and evolution of plant genomes. Annu Rev Plant Biol.

[32] Liu C., Cheng Y.J., Wang J.W., Weigel D. (2017). Prominent topologically associated domains differentiate global chromatin packing in rice from *Arabidopsis*. Nat Plants.

[33] Wang C.M., Liu C., Roqueiro D., Grimm D., Schwab R., Becker C. (2015). Genome-wide analysis of local chromatin packing in *Arabidopsis thaliana*. Genome Res.

[34] Song C., Liu Y.F., Song A.P., Dong G.Q., Zhao H.B., Sun W. (2018). The *Chrysanthemum nankingense* genome provides insights into the evolution and diversification of chrysanthemum flowers and medicinal traits. Mol Plant.

[35] Wang L.H., Yu S., Tong C.B., Zhao Y.Z., Liu Y., Song C. (2014). Genome sequencing of the high oil crop sesame provides insight into oil biosynthesis. Genome Biol.

[36] Chiang Y.C., Huang B.H., Liao P.C. (2012). Diversification, biogeographic pattern, and demographic history of Taiwanese *Scutellaria* species inferred from nuclear and chloroplast DNA. PLoS One.

[37] Jaillon O., Aury J.M., Noel B., Policriti A., Clepet C., Casagrande A. (2007). The grapevine genome sequence suggests ancestral hexaploidization in major angiosperm phyla. Nature.

[38] Murat F., Armero A., Pont C., Klgeneopp C., Salse J. (2017). Reconstructing the genome of the most recent common ancestor of flowering plants. Nat Genet.

[39] Navarro A., Barton N.H. (2003). Chromosomal speciation and molecular divergence–accelerated evolution in rearranged chromosomes. Science.

[40] Zhang X., Abrahan C., Colquhoun T.A., Liu C.J. (2017). A proteolytic regulator controlling chalcone synthase stability and flavonoid biosynthesis in *Arabidopsis*. Plant Cell.

[41] Zhang M., Zhang Y., Scheuring C.F., Wu C.C., Dong J.J., Zhang H.B. (2012). Preparation of megabase-sized DNA from a variety of organisms using the nuclei method for advanced genomics research. Nat Protoc.

[42] Koren S., Walenz B.P., Berlin K., Miller J.R., Bergman N.H., Phillippy A.M. (2017). Canu: scalable and accurate long-read assembly via adaptive *k*-mer weighting and repeat separation. Genome Res.

[43] Simao F.A., Waterhouse R.M., Ioannidis P., Kriventseva E.V., Zdobnov E.M. (2015). BUSCO: assessing genome assembly and annotation completeness with single-copy orthologs. Bioinformatics.

[44] Li H., Durbin R. (2009). Fast and accurate short read alignment with Burrows-Wheeler transform. Bioinformatics.

[45] Servant N., Varoquaux N., Lajoie B.R., Viara E., Chen C.J., Vert J.P. (2015). HiC-Pro: an optimized and flexible pipeline for Hi-C data processing. Genome Biol.

[46] Cantarel B.L., Korf I., Robb S.M., Parra G., Ross E., Moore B. (2008). MAKER: an easy-to-use annotation pipeline designed for emerging model organism genomes. Genome Res.

[47] Ghosh S., Chan C.K. (2016). Analysis of RNA-seq data using TopHat and Cufflinks. Methods Mol Biol.

[48] Emms D.M., Kelly S. (2019). OrthoFinder: phylogenetic orthology inference for comparative genomics. Genome Biol.

[49] De Bie T., Cristianini N., Demuth J.P., Hahn M.W. (2006). CAFE: a computational tool for the study of gene family evolution. Bioinformatics.

[50] Wang Y., Tang H., Debarry J.D., Tan X., Li J., Wang X. (2012). MCScanX: a toolkit for detection and evolutionary analysis of gene synteny and collinearity. Nucleic Acids Res.

[51] Van de Peer Y., Mizrachi E., Marchal K. (2017). The evolutionary significance of polyploidy. Nat Rev Genet.

[52] El-Gebali S., Mistry J., Bateman A., Eddy S.R., Luciani A., Potter S.C. (2019). The Pfam protein families database in 2019. Nucleic Acids Res.

[53] Kumar S., Stecher G., Li M., Knyaz C., Tamura K. (2018). MEGA X: molecular evolutionary genetics analysis across computing platforms. Mol Biol Evol.

[54] Wang Y.Q., Song F.H., Zhu J.W., Zhang S.S., Yang Y.D., Chen T.T. (2017). GSA: genome sequence archive. Genomics Proteomics Bioinformatics.

