# Supplementary material for: Comparative Genome Analysis of Scutellaria baicalensis and Scutellaria barbata Reveals the Evolution of Active Flavonoid Biosynthesis
Source: Genomics Proteomics Bioinformatics. 2020 Nov 4;18(3):230–40. doi: 10.1016/j.gpb.2020.06.002 (PMC7801248; doi:10.1016/j.gpb.2020.06.002)
Supplement: Supplementary File S1 — Supplementary methods and results. [file mmc1.docx]

**File S1 Supplementary methods and results**

1. **Genome sequencing and assemblies**
   1. **Next-generation sequencing for DNA and RNA**

Young leaves from *Scutellaria baicalensis* (*S. baicalensis*) and *Scutellaria barbata* (*S. barbata*) were collected to extract DNA and construct libraries for whole-genome shotgun sequencing. The root, stem, leaf, and flower tissues of *S. baicalensis* and *S. barbata* were collected and frozen for RNA extraction and library preparation. Three replicates of high-quality RNA were reverse transcribed, and the cDNA was fragmented and constructed using Illumina sequencing libraries. The short-insert paired-end libraries (DNA or cDNA, 250 bp or 500 bp) of *S. baicalensis* and *S. barbata* were sequenced on the Illumina HiSeq 4000 platform. The raw data were filtered to remove adapters and low-quality reads using Trimmonmatic (v0.36). In total, 48,850,109,484 and 39,808,157,990 clean genomic DNA reads of *S. baicalensis* and *S. barbata* were produced, respectively. In addition, 76,576,534,792 and 79,641,451,065 RNA-Seq reads from three replicates of root, stem, leaf and flower tissues of *S. baicalensis* and *S. barbata*, respectively, were obtained. The clean read data from the cDNA libraries were first assembled using Trinity, and 11,734 and 14,900 protein-coding genes were annotated in *S. baicalensis* and *S. barbata*, respectively, to assist the genome annotation.

- 1. **Estimation of genome size**

The genome size of *S. baicalensis* was estimated by flow cytometry (BD FACSCalibur, Franklin Lakes, NJ). The OTTO 1 nucleus extracting solution was used to extract nuclei, and propidium iodide was used as the fluorochrome. The *S. baicalensis* genome size was predicted to be 440.2 ± 10 Mb (±SD) when *Salvia miltiorrhiza* (585 Mb) data were used as internal standards (Figure S1A). The genome survey was performed via Genome Characteristics Estimation (GCE) using Illumina paired-end sequencing data as the input. The *k*-mer size was set as 21 (Figure S1B). The genome sizes of *S. baicalensis* and *S. barbata* were calculated to be 441.9 Mb and 404.6 Mb, respectively. The genome of *S. baicalensis* showed high heterozygosity of 0.96%, and *S. barbata* genome owned low heterozygosity of 0.28% (Figure S1).

- 1. **PacBio and ONT sequencing**

To produce the contiguous genome assembly, we performed Oxford Nanopore technology (ONT) sequencing for *S. baicalensis* and PacBio single molecule, real-time sequencing (SMRT) sequencing for *S. barbata*. In total, 10 flow cells from the ONT GridION X5 platform produced 52,035,200,672 bp raw reads with an N50 of 16.3 kb, and 15 SMRT cells from the Sequel platform generated 51,672,515,843 bp raw reads with an N50 of 9.8 kb. The raw data from the ONT and PacBio platforms were filtered using MinKNOW and SMRT Link, respectively. Then, the filtered reads were corrected using Canu packages with the following parameters. The statistics of the corrected reads are listed in Table S1.

*Canu correction*

> canu -correct -d huangqin -p huangqin -fast genomeSize = 440 m -nanopore-raw huangqin_raw.fastq

> canu -correct -d banzhilian -p banzhilian -fast genomeSize = 404 m -pacbio-raw banzhilian_raw.fastq

- 1. **Genome assemblies for *S. baicalensis* and *S. barbata***

The corrected long reads were assembled using SMARTdenovo as follows.

*SMARTdenovo*

> perl smartdenovo.pl -c 1 -t 36 -k 17 -p huangqin_smartdenovo huagnqin.correctedReads.fasta > smartdenovo.make

> make -f smartdenovo.make

The same method was performed for *S. barbata*.

Then, a 369.81 Mb draft genome with 419 contigs and an N50 of 2.07 Mb for *S. baicalensis* and a 353.11 Mb genome with 534 contigs and an N50 of 2.50 Mb for *S. barbata* were assembled using SMARTdenovo. Because of the insertion/deletion errors of single molecular sequencing, we employed Pilon to polish the initial SMARTdenovo assemblies three times using the gold standard of Illumina sequencing short reads. Finally, a 376.97 Mb genome with 419 contigs and an N50 of 2.10 Mb for *S. baicalensis* and a 352.95 Mb genome with 534 contigs and an N50 of 2.50 Mb for *S. barbata* were generated. According to the polishing procedures, PacBio sequencing produced more insertion errors than ONT; conversely, ONT produced more deletion errors than PacBio.

*3 × Pilon*

>bwa index huangqin.smratdenovo.fasta

bwa mem -t 48 huangqin.smratdenovo.fasta 250_R1_clean.fastq 250_R2_clean.fastq > 250_pilon.sam

samtools view -bhS -o 250_pilon.bam 250_pilon.sam

samtools sort 250_pilon.bam 250_sorted_pilon

samtools index 250_sorted_pilon.bam

…..

java -Xmx500G -jar pilon-1.22.jar --genome huagnqin.smratdenovo.fasta --frags 250_sorted_pilon1.bam --frages … --jumps… --output huangqin.smratdenovo_polish_pilon1.fas --threads 48

This step was repeated three times. The same method was performed for *S. barbata*.

- 1. **Chromosome-level assemblies for *S. baicalensis* and *S. barbata***

The contigs of the draft genomes were anchored to chromosomes using Hi-C technology. First, different numbers of chromosomes of *S. baicalensis* (2n = 18) and *S. barbata* (2n = 26) were detected. The young leaves of *S. baicalensis* and *S. barbata* were fixed, and DNA was crosslinked and cut using Hind III. The sticky ends of the Hind III cutting were repaired and biotinylated. Then, the blunt ends were ligated to form circles and fragmented, selected and used to construct sequencing libraries. Here, we generated 60,491,205,402 bp and 66,240,627,424 bp Hi-C reads of *S. baicalensis* and *S. barbata*, which covered the genomes more than 100 ×. The sequenced reads were aligned to the *S. baicalensis* and *S. barbata* genomes, and the unique paired alignments (100,093,998 and 130,543,934) of both genomes were selected. Among them, 84.73% (84,810,817) and 86.68% (113,149,190) of the alignments were valid interaction pairs for *S. baicalensis* and *S. barbata*, respectively. The chromosome numbers, enzyme cutting sites, draft genomes and valid Hi-C reads were set in the LACHESIS script. Lastly, 99.8% and 98.8% of the assembled *S. baicalensis* and *S. barbata* contigs were corrected and anchored to 9 and 13 pseudochromosomes (2n = 18 for *S. baicalensis*, 2n = 26 for *S. barbata*) using a Hi-C interaction matrix with N50 values of 40.8 Mb and 23.7 Mb, respectively. The Hi-C intrachromosomal contact maps of the *S. baicalensis* and *S. barbata* chromosomes are shown in Figure S2. The assembled statistics are listed in Table S2. The genome collinearity between *S. baicalensis* and *S. barbata* was determined using MCScan and BLASTN (Figure S3, Table S3), indicating the chromosomal rearrangements of sister species.

1. **Genome annotation**

The *S. baicalensis* genome comprised 33,414 protein-coding genes and 2833 noncoding RNAs (ncRNAs), and 41,697 genes and 1768 ncRNAs were annotated in the *S. barbata* genome using MAKER and Infernal (Table S4). The assembled transcripts and predicted amino acids from the RNA-Seq data of *S. baicalensis* and *S. barbata* and the protein sequences of *Arabidopsis thaliana* were used to assist the *ab initio* annotation. The transposable element (TE) annotation and long terminal repeat (LTR) insertion time were further analyzed using RepeatModeler, RepeatMasker, LTR_Finder (v1.0.6) and LTR_retriever, and the results are shown in Tables S5–S7 and Figure S4.

There were almost four times more rRNAs in *S. baicalensis* (813) than in *S. barbata* (210), and the 8 s RNA of *S. baicalensis* presented significant expansion. The 18 s RNA and 28 s RNA from *S. baicalensis* were almost localized in chr7 (Sbai7); however, these rRNAs from *S. barbata* were distributed in different chromosomes (Table S8).

1. **Genome evolution and whole-genome duplication**

Orthologous groups from 9 angiosperms were analyzed using OrthoMCL (v 2.0.9), and a total of 31,205 orthologous groups covering 265,507 coding genes were identified. The unique and common genes among the tested plants were classified and annotated using the PFAM database. Among the orthologous groups, 726 single-copy genes in all tested plants were identified. Here, we aligned 100 single-copy genes and constructed a phylogenetic tree for the tested angiosperms. The divergence time of each species was calculated using r8s based on the reported divergence time of *Brachypodium distachyon*-*Oryza sativa* (47 million years ago [MYA]) and *Populus trichocarpa*-*Arabidopsis thaliana* (106 MYA). The results indicated that the speciation time of *S. baicalensis* and *S. barbata* was 14.24 MYA. According to the phylogenetic tree and divergence times, the expansion and contraction of gene families among the 9 angiosperms were calculated using CAFÉ (Figure S7A, Table S10).

The *Ks* (synonymous substitution rate) value was calculated for *S. baicalensis*–*S. baicalensis*, *S. barbata*–*S. barbata*, *S. miltiorrhiza*–*S. miltiorrhiza*, *S. baicalensis*–*S. miltiorrhiza*, *S. baicalensis*–*S. barbata*, and *S. barbata*–*S. miltiorrhiza* to evaluate WGD events. The 17,971, 9948, and 11,078 paralogous gene pairs of *S. baicalensis*–*S. barbata*, *S. barbata*–*S. miltiorrhiza*, and *S. baicalensis*–*S. miltiorrhiza* were identified, and their *Ks* peaks were 0.17, 0.61, and 0.61, corresponding to divergence times of 14.24, 51.35, 51.35 MYA, respectively. In addition, 14,058, 14,422, and 16,172 orthologous gene pairs of *S. miltiorrhiza*, *S. baicalensis*, and *S. barbata* were identified, with *Ks* peaks of 1.02, 0.89, and 0.86, suggesting that these three species of Lamiaceae shared a whole-genome duplication (WGD) event, which occurred between 72.04 and 85.44 MYA (Figure S7B and C).

1. **Identification of flavonoid contents in different tissues of *S. baicalensis* and *S. barbata***

Different tissues, namely, the root, stem, leaf, and flower tissues, of *S. baicalensis* and *S. barbata* were collected and dried at 45°C. Then, the samples were pulverized into homogeneous powders (40 mesh). The dried powder (0.1 g), which was weighed accurately, was extracted with 1 mL 60% ethanol. After accurate weighing, ultrasonication (100 kHz) was performed at room temperature for 60 min, and the same solvent was added to compensate for the weight lost during extraction and filtered through a 0.22-μm membrane for ultraperformance liquid chromatography (UPLC) analysis.

Analysis was performed on a Thermo Ultimate3000 (UHPLC) system consisting of a quaternary pump solvent management system, an online degasser, an autosampler, and a DAD detector. An Acquity UPLC BEH C18 (2.1 mm × 100 mm, 1.7 μm) column was applied for all analyses. The mobile phase was composed of A (water and 0.1% formic acid) and B (acetonitrile and 0.1% formic acid) using a gradient elution of 5%−10% B at 0−10 min, 10%−15% B at 10−15 min, 15% B at 15−20 min, 15%−50% B at 20−40 min, 50%−100% B at 40−42 min, 100% B at 42−44 min. The flow rate of the mobile phase was set at 0.300 ml/min. The column temperature was conditioned at 30 °C, and the autosampler was maintained at 10 °C. The injection volume of the root tissue of *S. baicalensis* was 2 μl, and other injection volumes were 5 μl. The results are shown in Figure S8 and Table S16.
