# Supplementary material for: Comparative Genome Analysis of Scutellaria baicalensis and Scutellaria barbata Reveals the Evolution of Active Flavonoid Biosynthesis
Source: Genomics Proteomics Bioinformatics. 2020 Nov 4;18(3):230–40. doi: 10.1016/j.gpb.2020.06.002 (PMC7801248; doi:10.1016/j.gpb.2020.06.002)
Supplement: Supplementary Figure S1 — Genome size estimation. A. Flow cytometry analysis using Salvia miltiorrhiza data as internal standards. The number of nuclei counted in tested samples is indicated on the Y axis. The relative fluorescence of FL2-A (585/40 nm) is displayed on the X axis. B. The 21 k-mer distribution from Illumina short reads of S. baicalensis and S. barbata. [file mmc2.pptx]

## Slide 1
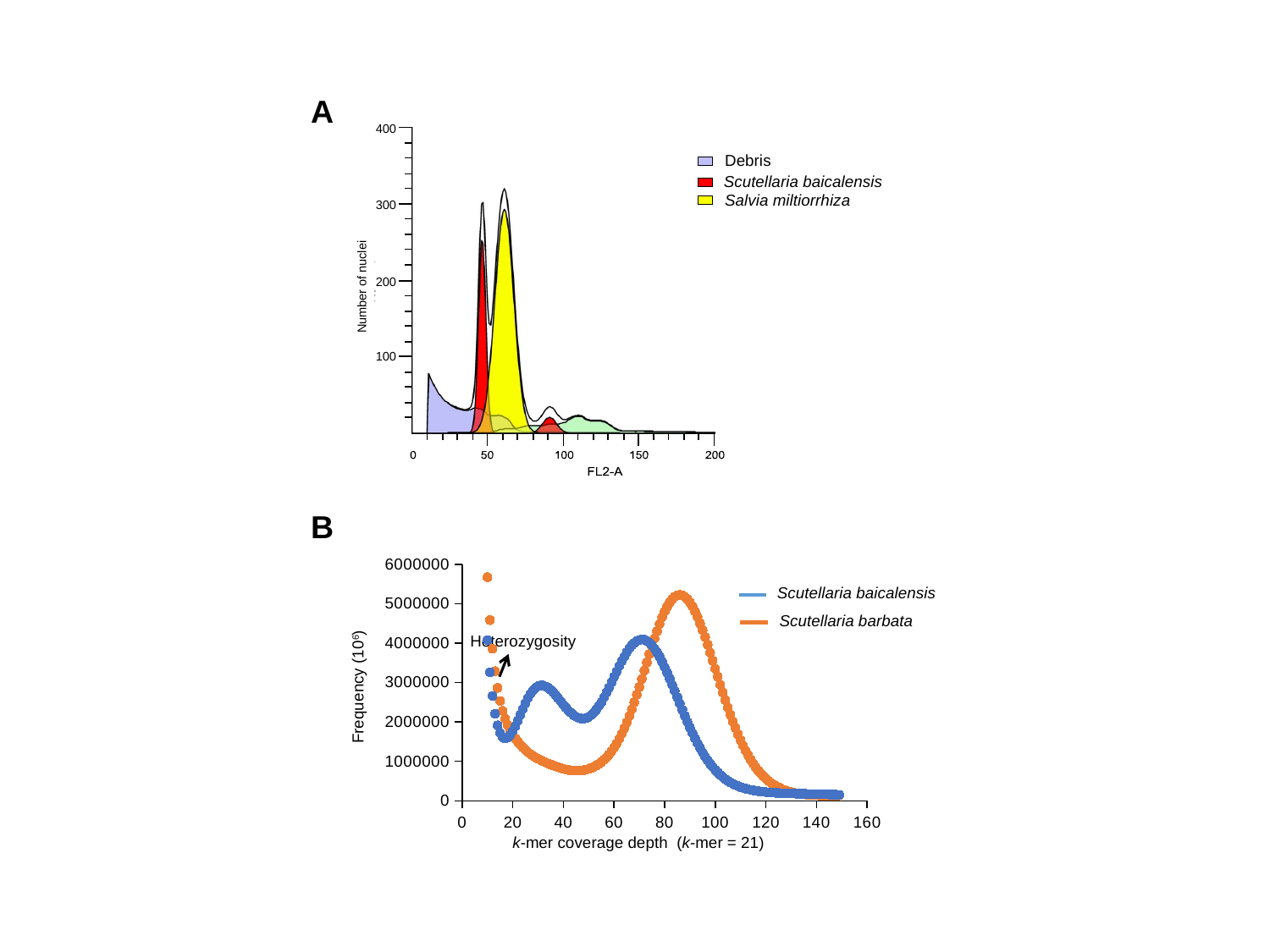

A
400
Debris
Scutellaria baicalensis
Salvia miltiorrhiza
300
200
Number of nuclei
100
B
### Chart
| Category | | |
|---|---|---|Scutellaria baicalensis
Scutellaria barbata
Heterozygosity
Frequency (106)
k-mer coverage depth (k-mer = 21)
