# Supplementary material for: Comparative Genome Analysis of Scutellaria baicalensis and Scutellaria barbata Reveals the Evolution of Active Flavonoid Biosynthesis
Source: Genomics Proteomics Bioinformatics. 2020 Nov 4;18(3):230–40. doi: 10.1016/j.gpb.2020.06.002 (PMC7801248; doi:10.1016/j.gpb.2020.06.002)
Supplement: Supplementary Figure S2 — Hi-C intrachromosomal contact map. The red diagonal line indicates a high number of intrachromosomal contacts. A. Hi-C heatmap of S. baicalensis. Number of links at the 100-kb resolution is indicated. B. Hi-C heatmap of S. barbata. Hi-C, high-through chromosome conformation capture. [file mmc3.pptx]

## Slide 1
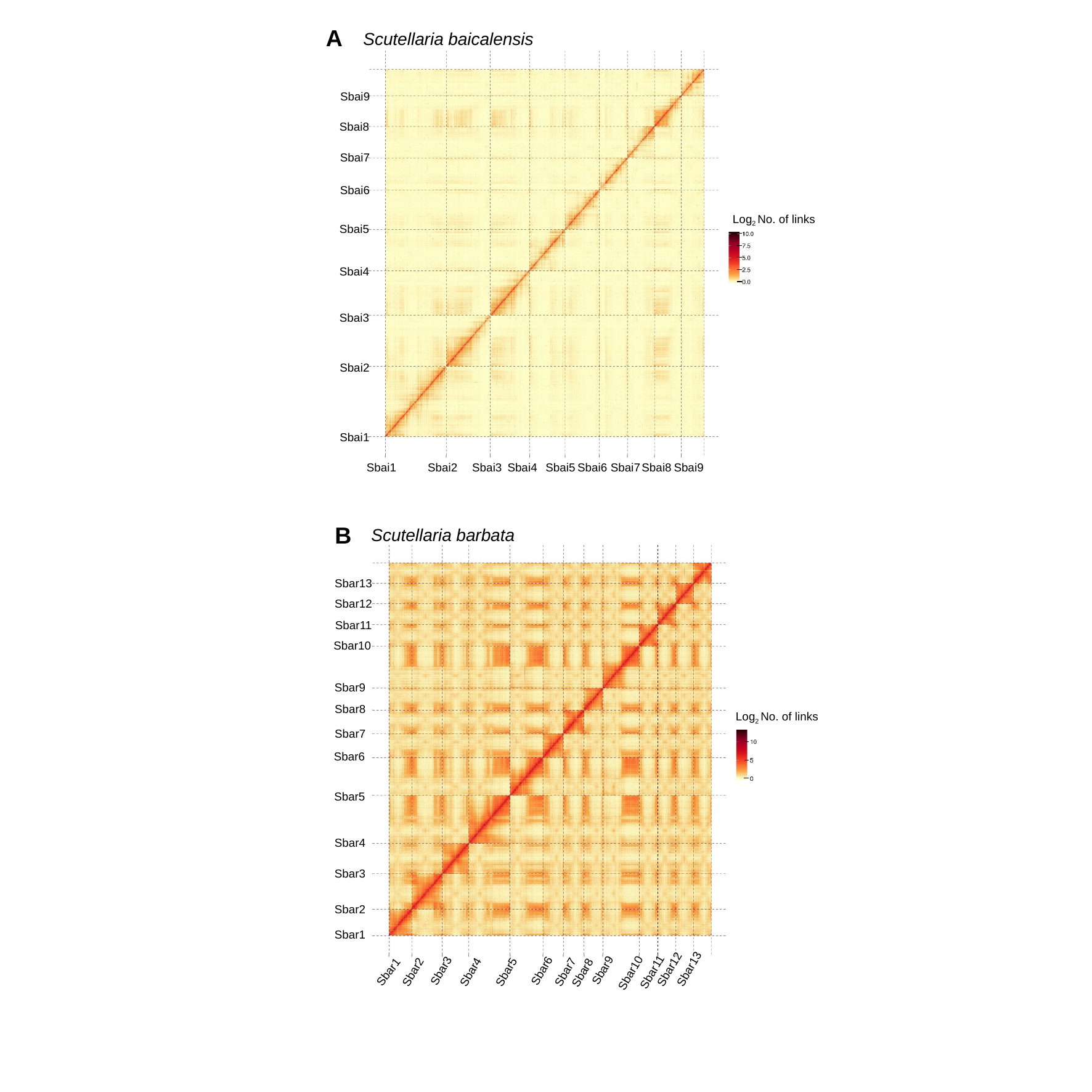

A
Scutellaria baicalensis
Sbai9
Sbai8
Sbai7
Sbai6
Log2 No. of links
Sbai5
Sbai4
Sbai3
Sbai2
Sbai1
Sbai2
Sbai3
Sbai4
Sbai5
Sbai6
Sbai7
Sbai8
Sbai9
Sbai1
B
Scutellaria barbata
Sbar13
Sbar12
Sbar11
Sbar10
Sbar9
Sbar8
Log2 No. of links
Sbar7
Sbar6
Sbar5
Sbar4
Sbar3
Sbar2
Sbar1
Sbar13
Sbar12
Sbar9
Sbar3
Sbar6
Sbar1
Sbar2
Sbar11
Sbar4
Sbar5
Sbar7
Sbar8
Sbar10
