# Supplementary material for: Comparative Genome Analysis of Scutellaria baicalensis and Scutellaria barbata Reveals the Evolution of Active Flavonoid Biosynthesis
Source: Genomics Proteomics Bioinformatics. 2020 Nov 4;18(3):230–40. doi: 10.1016/j.gpb.2020.06.002 (PMC7801248; doi:10.1016/j.gpb.2020.06.002)
Supplement: Supplementary Figure S3 — Genome synteny analysis. The genome synteny results were analyzed using MCScanX between S. baicalensis and S. barbata (A), S. baicalensis and S. indicum (B), S. barbata and S. indicum (C), respectively. [file mmc4.pptx]

## Slide 1
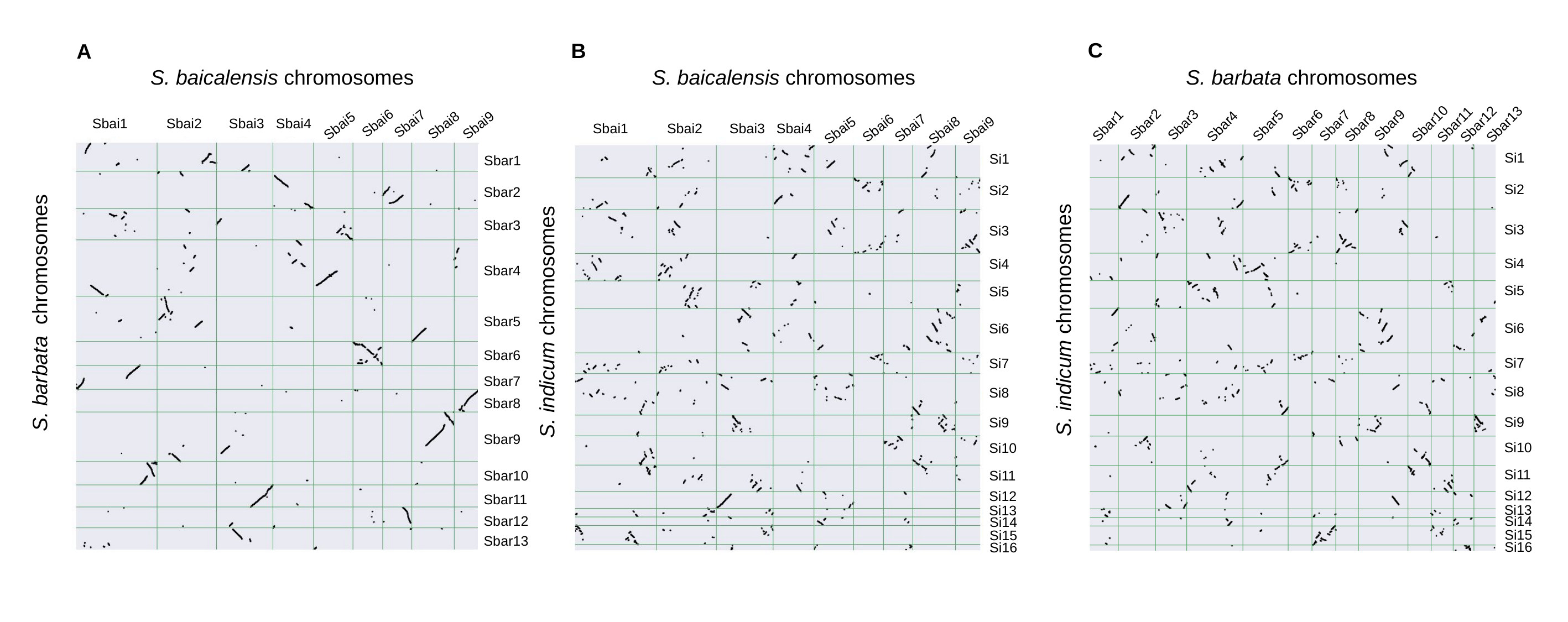

C
B
A
S. baicalensis chromosomes
S. barbata chromosomes
S. baicalensis chromosomes
S. indicum chromosomes
S. barbata chromosomes
S. indicum chromosomes
Sbai6
Sbar10
Sbai1 Sbai2 Sbai3 Sbai4
Sbai7
Sbar2
Sbar13
Sbar12
Sbar11
Sbar3
Sbar6
Sbar9
Sbai9
Sbai8
Sbar7
Sbar1
Sbar5
Sbai5
Sbar4
Sbar8
Sbai6
Sbai1 Sbai2 Sbai3 Sbai4
Sbai7
Sbai9
Sbai8
Sbai5
Si1
Si1
Sbar1
Si2
Si2
Sbar2
Sbar3
Si3
Si3
Si4
Si4
Sbar4
Si5
Si5
Sbar5
Si6
Si6
Sbar6
Si7
Si7
Sbar7
Si8
Si8
Sbar8
Si9
Si9
Sbar9
Si10
Si10
Si11
Si11
Sbar10
Si12
Si12
Sbar11
Si13
Si13
Sbar12
Si14
Si14
Si15
Si15
Sbar13
Si16
Si16
