# Supplementary material for: Comparative Genome Analysis of Scutellaria baicalensis and Scutellaria barbata Reveals the Evolution of Active Flavonoid Biosynthesis
Source: Genomics Proteomics Bioinformatics. 2020 Nov 4;18(3):230–40. doi: 10.1016/j.gpb.2020.06.002 (PMC7801248; doi:10.1016/j.gpb.2020.06.002)
Supplement: Supplementary Figure S4 — Alignment of large-scale DNA sequences. The dot plot presented the genome alignment between S. baicalensis and S. barbata using MUMmer with the minimum mapping length of 100 kb. The red dots represent forward matches, and the blue dots represent reverse matches. [file mmc5.pptx]

## Slide 1
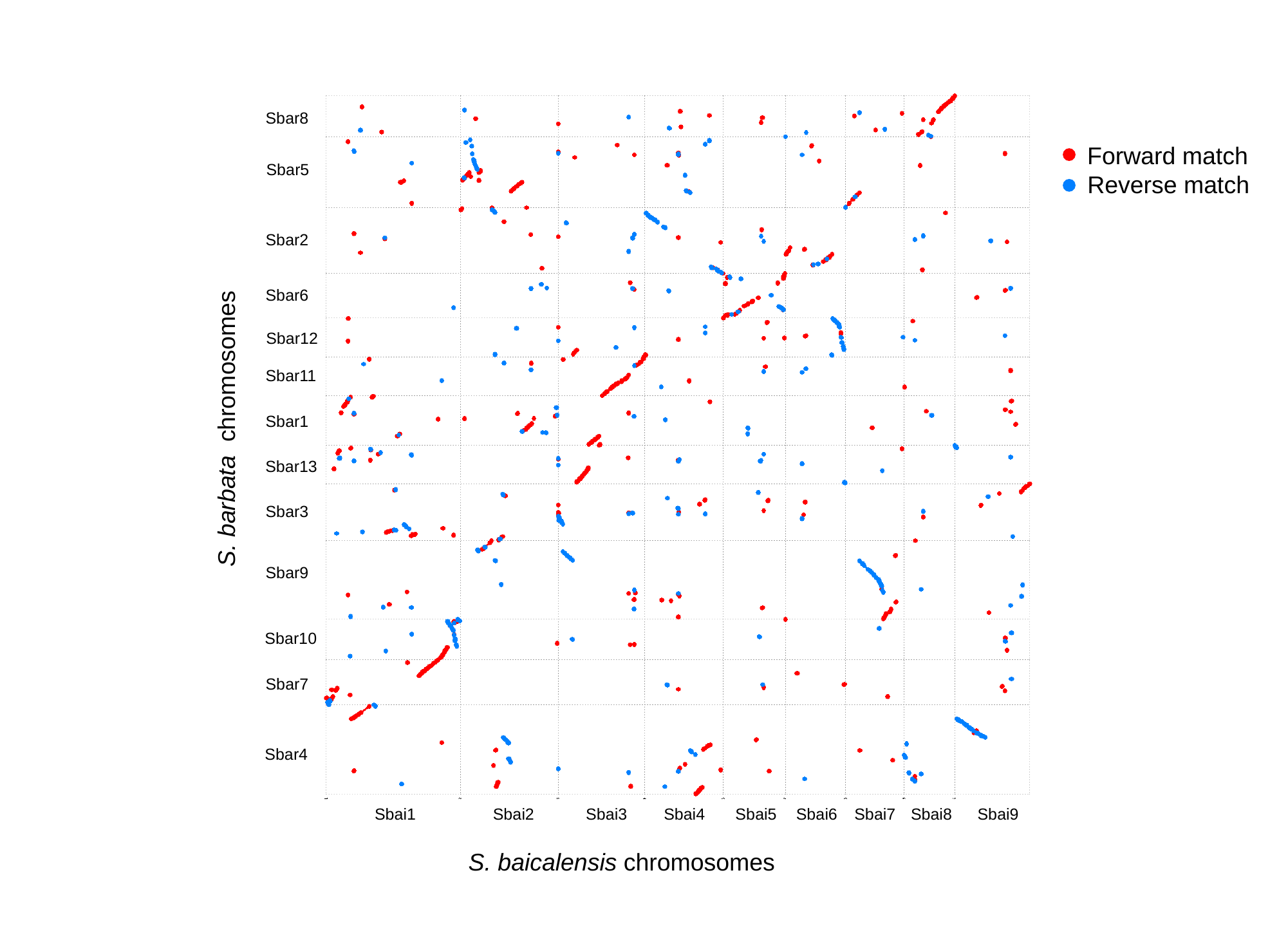

Sbai2
Sbai3
Sbai4
Sbai5
Sbai6
Sbai7
Sbai8
Sbai9
Sbai1
Sbar8
Sbar5
Sbar2
Sbar6
Sbar12
Sbar11
Sbar1
Sbar13
Sbar3
Sbar9
Sbar10
Sbar7
Sbar4
S. barbata chromosomes
S. baicalensis chromosomes
Forward match
Reverse match
