# Supplementary material for: Comparative Genome Analysis of Scutellaria baicalensis and Scutellaria barbata Reveals the Evolution of Active Flavonoid Biosynthesis
Source: Genomics Proteomics Bioinformatics. 2020 Nov 4;18(3):230–40. doi: 10.1016/j.gpb.2020.06.002 (PMC7801248; doi:10.1016/j.gpb.2020.06.002)
Supplement: Supplementary Figure S5 — Insertion time distribution of intact LTR-RTs. A. Difference of LTR-RT distribution between S. baicalensis and S. barbata. B. Difference of insertion time of LTR-RTs between S. baicalensis and S. barbata. LTR-RT, long terminal repeat retrotransposon. [file mmc6.pptx]

## Slide 1
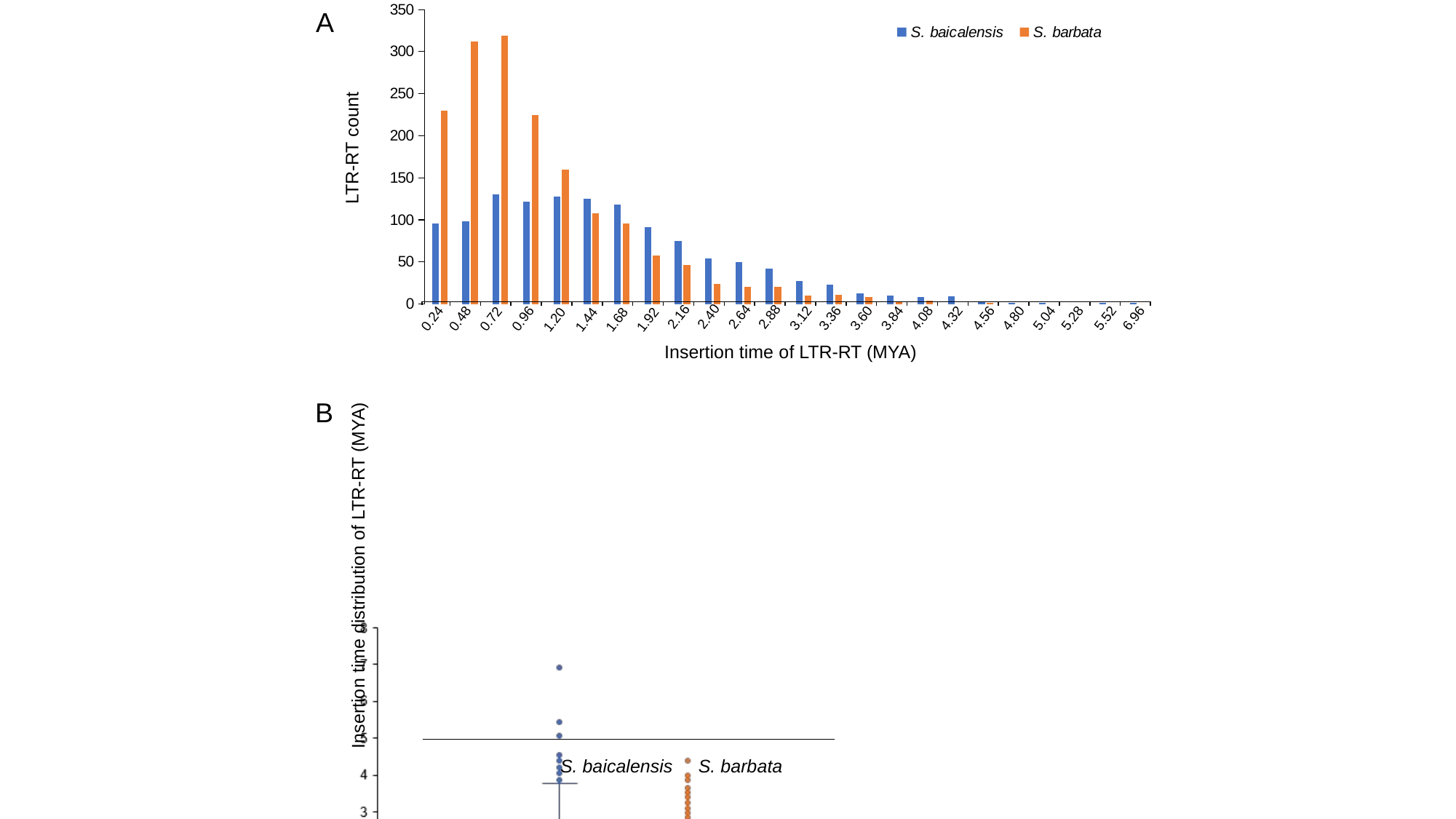

A
### Chart
| Category | S. baicalensis | S. barbata |
|---|---|---|
| 240000 | 96.0 | 230.0 |
| 480000 | 98.0 | 312.0 |
| 720000 | 130.0 | 319.0 |
| 960000 | 122.0 | 225.0 |
| 1200000 | 128.0 | 160.0 |
| 1440000 | 125.0 | 108.0 |
| 1680000 | 118.0 | 96.0 |
| 1920000 | 91.0 | 58.0 |
| 2160000 | 75.0 | 46.0 |
| 2400000 | 54.0 | 24.0 |
| 2640000 | 50.0 | 20.0 |
| 2880000 | 42.0 | 20.0 |
| 3120000 | 27.0 | 10.0 |
| 3360000 | 23.0 | 11.0 |
| 3600000 | 13.0 | 8.0 |
| 3840000 | 10.0 | 2.0 |
| 4080000 | 8.0 | 4.0 |
| 4320000 | 9.0 | 0.0 |
| 4560000 | 2.0 | 1.0 |
| 4800000 | 1.0 | 0.0 |
| 5040000 | 1.0 | 0.0 |
| 5280000 | 0.0 | 0.0 |
| 5520000 | 1.0 | 0.0 |
| 6960000 | 1.0 | 0.0 |LTR-RT count
2.40
2.88
2.64
2.16
6.96
5.52
5.28
5.04
4.56
4.80
3.84
4.08
4.32
3.60
3.12
3.36
0.96
0.24
0.48
0.72
1.20
1.68
1.92
1.44
Insertion time of LTR-RT (MYA)
B
S. baicalensis
S. barbata
Insertion time distribution of LTR-RT (MYA)
