# Supplementary material for: Comparative Genome Analysis of Scutellaria baicalensis and Scutellaria barbata Reveals the Evolution of Active Flavonoid Biosynthesis
Source: Genomics Proteomics Bioinformatics. 2020 Nov 4;18(3):230–40. doi: 10.1016/j.gpb.2020.06.002 (PMC7801248; doi:10.1016/j.gpb.2020.06.002)
Supplement: Supplementary Figure S6 — Genome-wide chromatin packing analysis in S. baicalensis. A. The intrachromosomal interactions revealing the A compartments (red boxes) and B (blue boxes) compartments of S. baicalensis. B. The ratio of TE and gene numbers between the A and B compartments. C. The interchromosomal interactions of S. baicalensis. The heatmap based on Log2(Obs/Exp) indicates that the chromatin interaction frequencies are transformed into normalized values. TE, transposable element; Obs, observed; Exp, expected. [file mmc7.pptx]

## Slide 1
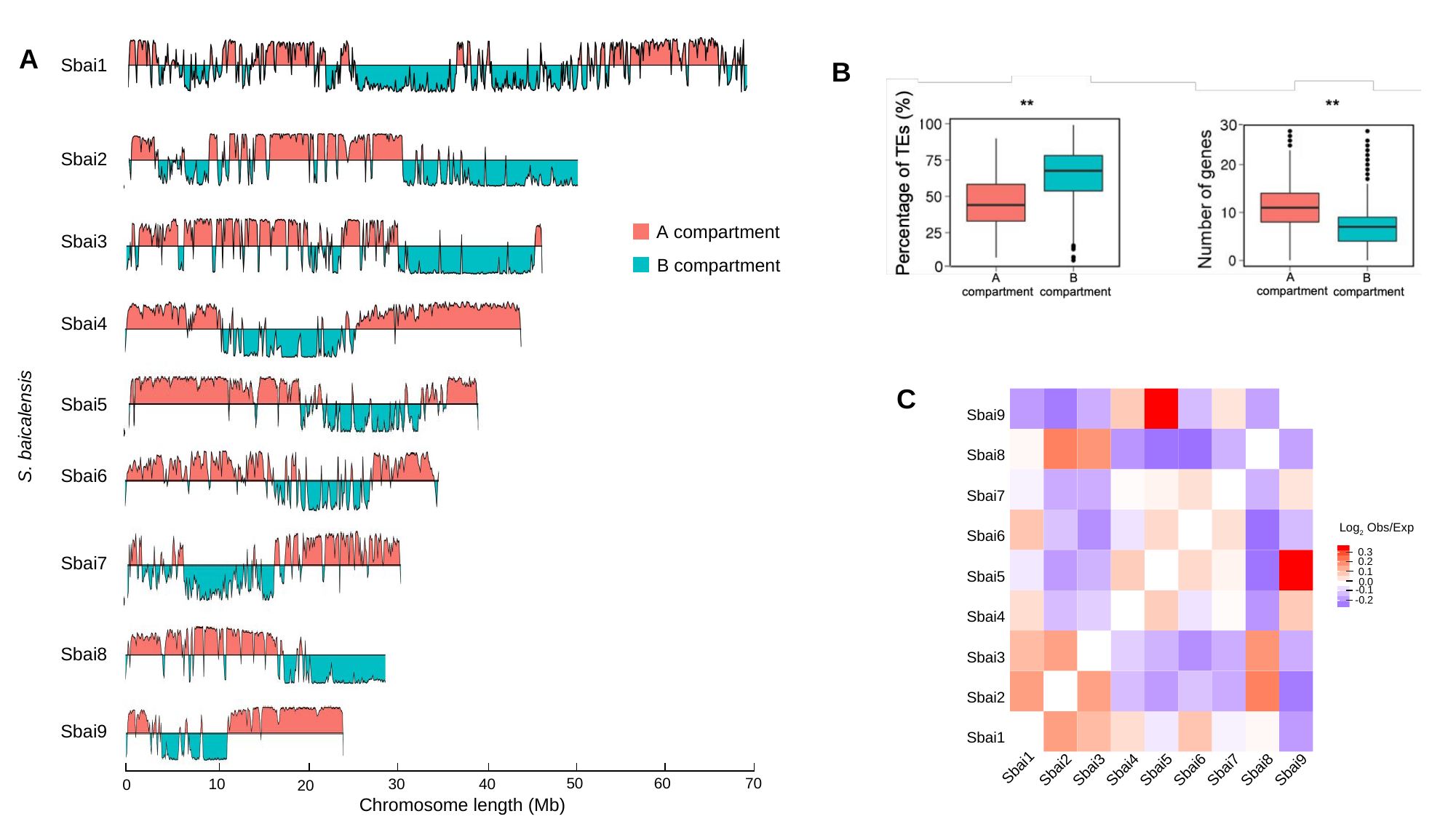

A
Sbai1
B
Sbai2
A compartment
Sbai3
B compartment
Sbai4
Sbai9
Sbai8
Sbai7
Log2(Obs/Exp)
Sbai6
0.3
0.2
0.1
Sbai5
0.0
-0.1
-0.2
Sbai4
Sbai3
Sbai2
Sbai1
Sbai1
Sbai2
Sbai3
Sbai4
Sbai5
Sbai6
Sbai7
Sbai8
Sbai9
C
Sbai5
S. baicalensis
Sbai6
Log2 Obs/Exp
Sbai7
Sbai8
Sbai9
70
60
50
10
40
30
0
20
Chromosome length (Mb)
