# Supplementary material for: Comparative Genome Analysis of Scutellaria baicalensis and Scutellaria barbata Reveals the Evolution of Active Flavonoid Biosynthesis
Source: Genomics Proteomics Bioinformatics. 2020 Nov 4;18(3):230–40. doi: 10.1016/j.gpb.2020.06.002 (PMC7801248; doi:10.1016/j.gpb.2020.06.002)
Supplement: Supplementary Figure S7 — Genome-wide chromatin packing analysis in S. barbata. A. The intrachromosomal interactions revealing the A (red boxes) and B (blue boxes) compartments of S. barbata. B. The ratio of TE and gene numbers between the A and B compartments. C. The interchromosomal interactions of S. barbata. The heatmap based on Log2(Obs/Exp) indicates that the chromatin interaction frequencies are transformed into normalized observed/expected values. TE, transposable element; Obs, observed; Exp, expected. [file mmc8.pptx]

## Slide 1
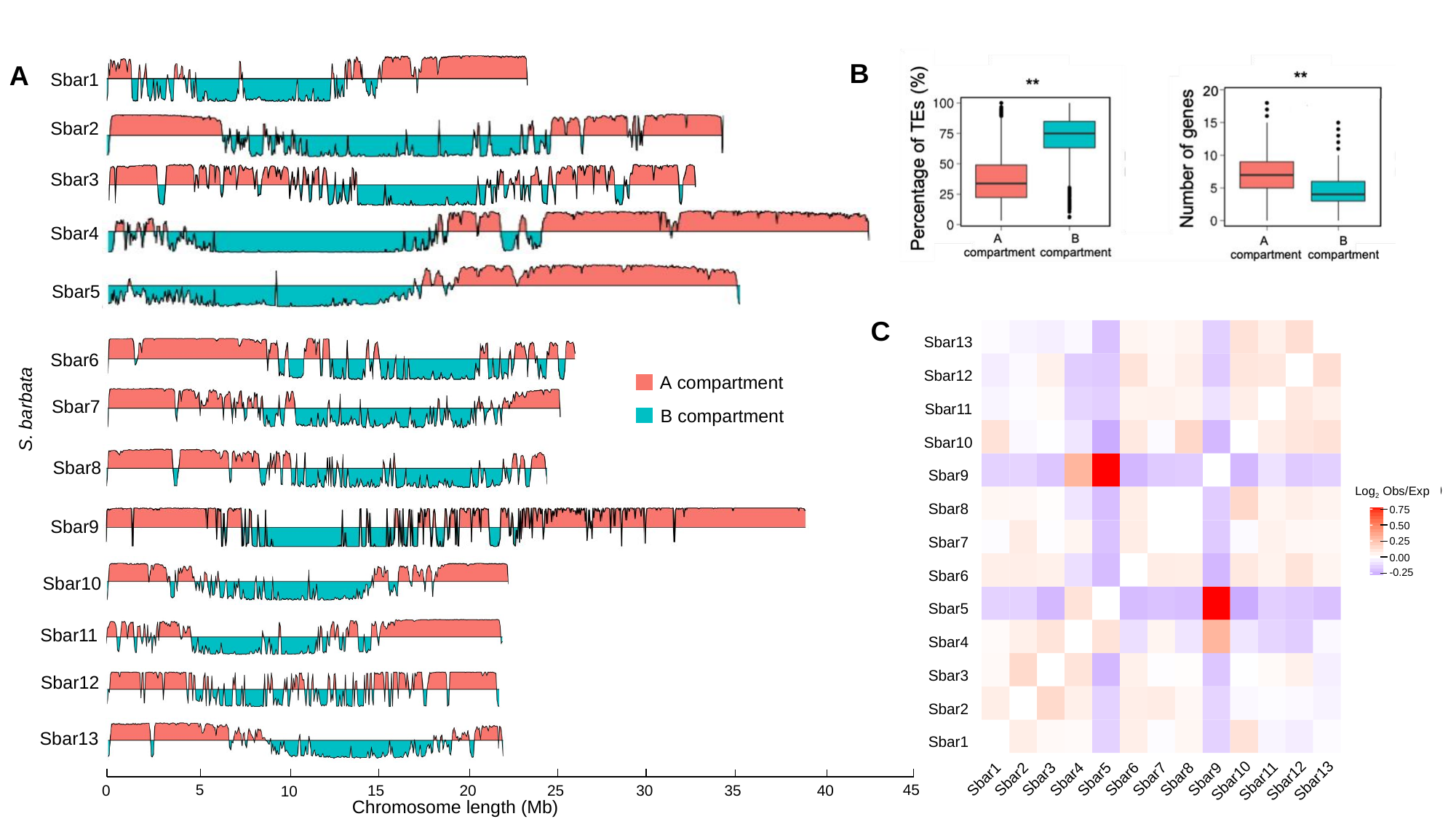

A
Sbar1
Sbar2
Sbar3
Sbar4
Sbar5
Sbar6
Sbar7
Sbar8
Sbar9
Sbar10
Sbar11
Sbar12
Sbar13
B
Sbar13
Sbar12
Sbar11
Sbar10
Sbar9
Log2(Obs/Exp)
Sbar8
0.75
0.50
Sbar7
0.25
0.00
-0.25
Sbar6
Sbar5
Sbar4
Sbar3
Sbar2
Sbar1
Sbar1
Sbar2
Sbar3
Sbar4
Sbar5
Sbar6
Sbar7
Sbar8
Sbar9
Sbar10
Sbar11
Sbar12
Sbar13
C
A compartment
S. barbata
B compartment
Log2 Obs/Exp
5
45
35
40
20
15
30
25
0
10
Chromosome length (Mb)
