# Supplementary material for: Comparative Genome Analysis of Scutellaria baicalensis and Scutellaria barbata Reveals the Evolution of Active Flavonoid Biosynthesis
Source: Genomics Proteomics Bioinformatics. 2020 Nov 4;18(3):230–40. doi: 10.1016/j.gpb.2020.06.002 (PMC7801248; doi:10.1016/j.gpb.2020.06.002)
Supplement: Supplementary Figure S8 — Gene family expansion and contraction. The numbers of each branch represent the gene family expansion (in red) and contraction (in green). The number of expansion, remain, and contraction events of 20 nodes is also listed in Table S10. [file mmc9.pptx]

## Slide 1
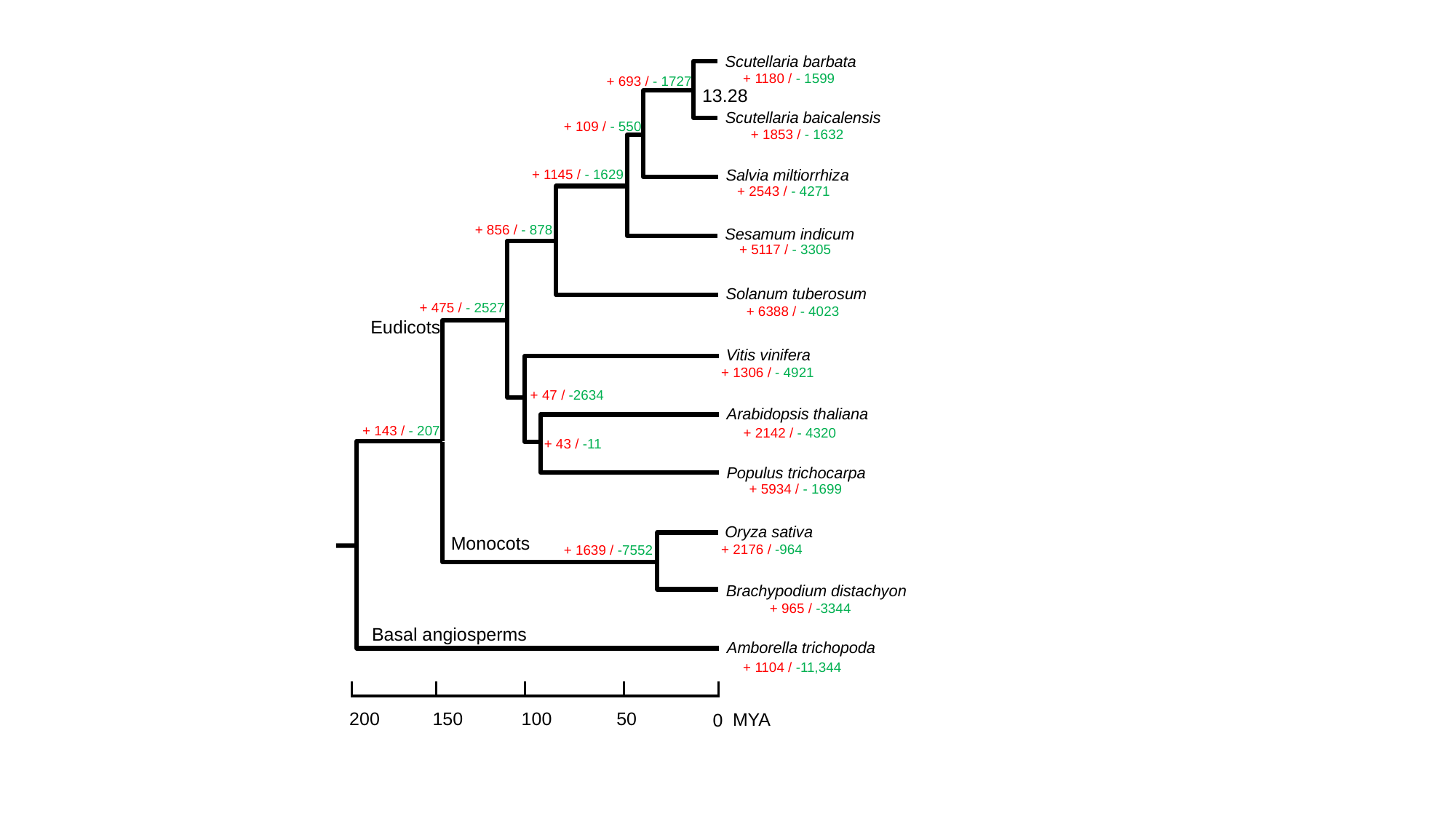

Scutellaria barbata
13.28
 Scutellaria baicalensis
 Salvia miltiorrhiza
 Sesamum indicum
 Solanum tuberosum
Eudicots
 Vitis vinifera
 Arabidopsis thaliana
 Populus trichocarpa
 Oryza sativa
Monocots
 Brachypodium distachyon
Basal angiosperms
 Amborella trichopoda
50
200
150
100
MYA
+ 1180 / - 1599
+ 693 / - 1727
+ 109 / - 550
+ 1853 / - 1632
+ 1145 / - 1629
+ 2543 / - 4271
+ 856 / - 878
+ 5117 / - 3305
+ 475 / - 2527
+ 6388 / - 4023
+ 1306 / - 4921
+ 47 / -2634
+ 143 / - 207
+ 2142 / - 4320
+ 43 / -11
+ 5934 / - 1699
+ 2176 / -964
+ 1639 / -7552
+ 965 / -3344
+ 1104 / -11,344
0
