# Supplementary material for: Comparative Genome Analysis of Scutellaria baicalensis and Scutellaria barbata Reveals the Evolution of Active Flavonoid Biosynthesis
Source: Genomics Proteomics Bioinformatics. 2020 Nov 4;18(3):230–40. doi: 10.1016/j.gpb.2020.06.002 (PMC7801248; doi:10.1016/j.gpb.2020.06.002)
Supplement: Supplementary Figure S9 — Synteny analysis against V. vinifera genome. The V. vinifera genome was painted into S. baicalensis and S. barbata genome, respectively, based on the gene collinearity using MCScanX. [file mmc10.pptx]

## Slide 1
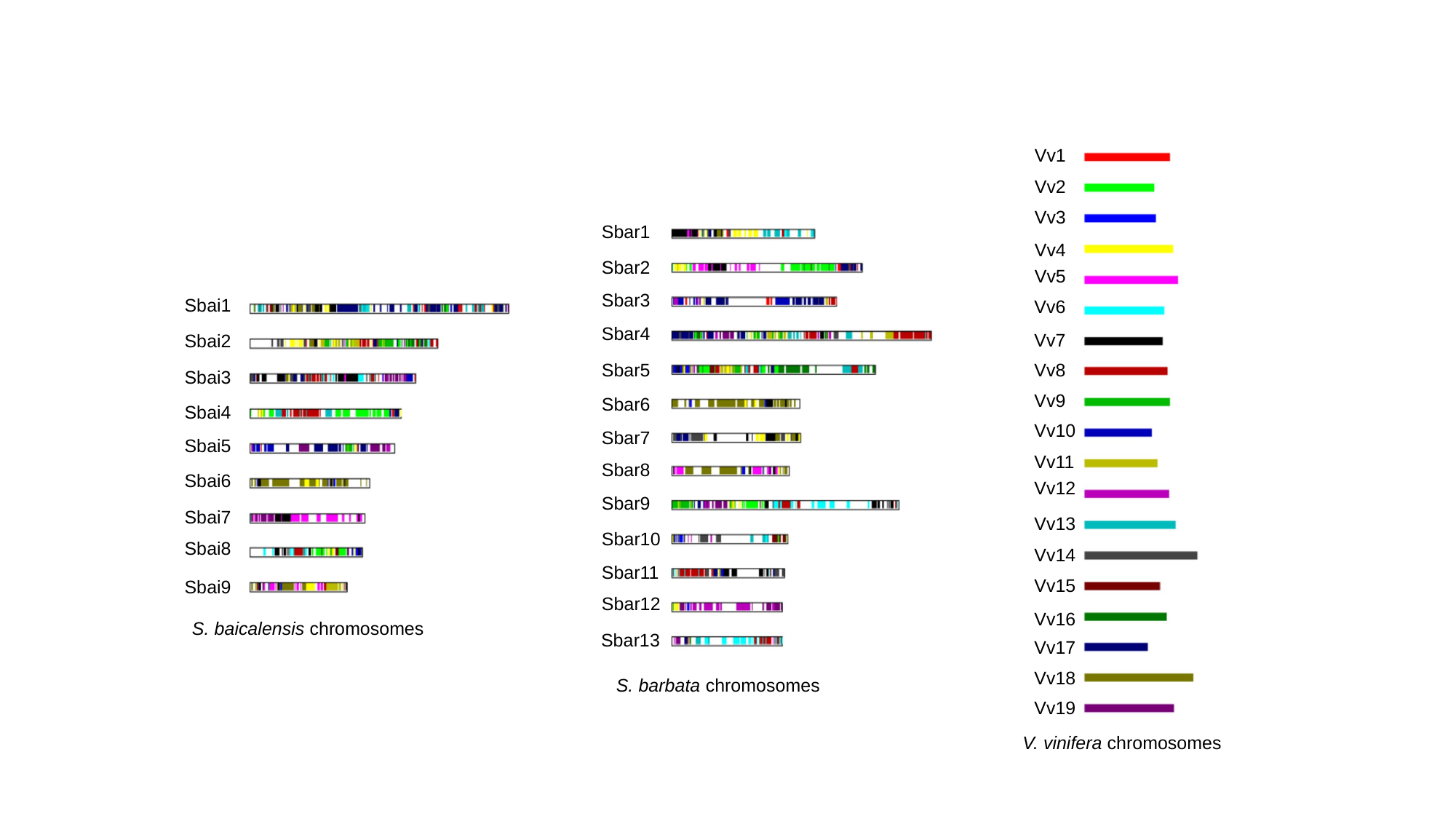

Vv1
Vv2
Vv3
Vv4
Vv5
Vv6
Vv7
Vv8
Vv9
Vv10
Vv11
Vv12
Vv13
Vv14
Vv15
Vv16
Vv17
Vv18
Vv19
Sbar1
Sbar2
Sbar3
Sbar4
Sbar5
Sbar6
Sbar7
Sbar8
Sbar9
Sbar10
Sbar11
Sbar12
Sbar13
Sbai1
Sbai2
Sbai3
Sbai4
Sbai5
Sbai6
Sbai7
Sbai8
Sbai9
S. baicalensis chromosomes
S. barbata chromosomes
V. vinifera chromosomes
