# Supplementary material for: Comparative Genome Analysis of Scutellaria baicalensis and Scutellaria barbata Reveals the Evolution of Active Flavonoid Biosynthesis
Source: Genomics Proteomics Bioinformatics. 2020 Nov 4;18(3):230–40. doi: 10.1016/j.gpb.2020.06.002 (PMC7801248; doi:10.1016/j.gpb.2020.06.002)
Supplement: Supplementary Figure S10 — Genome synteny analysis among related species. Dot plots presented the gene synteny between V. vinifera and S. indicum (A), V. vinifera and S. baicalensis (B), V. vinifera and S. barbata (C), respectively. The red circles highlighted the duplication events after WGT-γ event. Dot plots of paralogs in S. indicum (D), S. baicalensis (E), and S. barbata (F) to show the potential duplication events. [file mmc11.pptx]

## Slide 1
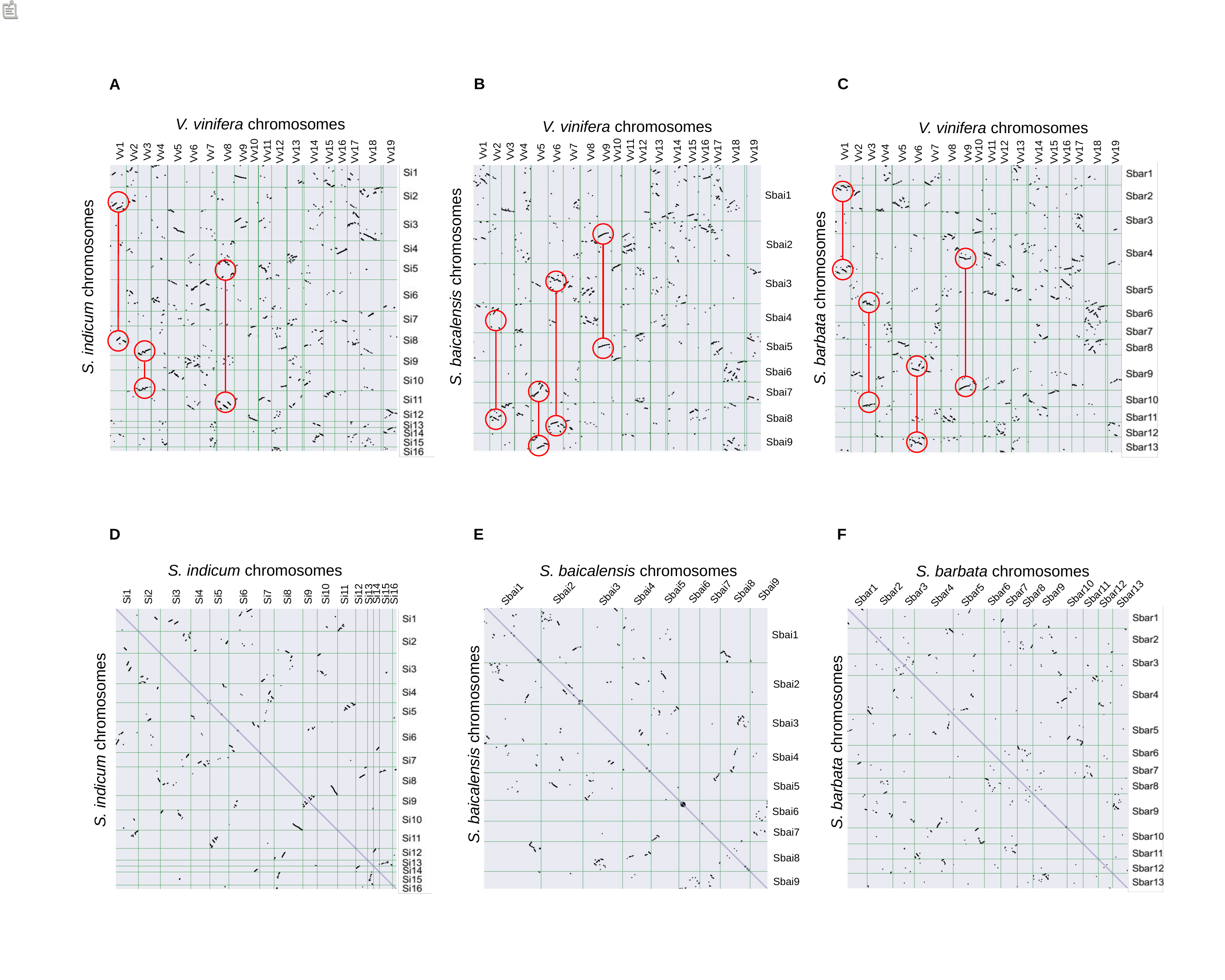

B
C
A
V. vinifera chromosomes
S. indicum chromosomes
V. vinifera chromosomes
S. baicalensis chromosomes
V. vinifera chromosomes
S. barbata chromosomes
Vv1
Vv3
Vv10
Vv11
Vv2
Vv4
Vv5
Vv12
Vv13
Vv14
Vv15
Vv16
Vv17
Vv18
Vv19
Vv6
Vv8
Vv7
Vv9
Vv1
Vv3
Vv10
Vv11
Vv2
Vv4
Vv5
Vv12
Vv13
Vv14
Vv15
Vv16
Vv17
Vv18
Vv19
Vv6
Vv8
Vv7
Vv9
Vv1
Vv3
Vv10
Vv11
Vv2
Vv4
Vv5
Vv12
Vv13
Vv14
Vv15
Vv16
Vv17
Vv18
Vv19
Vv6
Vv8
Vv7
Vv9
Sbai1
Sbai2
Sbai3
Sbai4
Sbai5
Sbai6
Sbai7
Sbai8
Sbai9
E
F
D
S. indicum chromosomes
S. indicum chromosomes
S. baicalensis chromosomes
S. barbata chromosomes
S. barbata chromosomes
S. baicalensis chromosomes
Sbar10
Sbar2
Sbar13
Sbar12
Sbar11
Sbar3
Sbar6
Sbar9
Sbar7
Sbar1
Sbar5
Sbar4
Sbar8
Si14
Si15
Si16
Si12
Si13
Si10
Si11
Si1
Si2
Si3
Si4
Si5
Si6
Si7
Si8
Si9
Sbai9
Sbai8
Sbai6
Sbai7
Sbai5
Sbai2
Sbai1
Sbai4
Sbai3
Sbai1
Sbai2
Sbai3
Sbai4
Sbai5
Sbai6
Sbai7
Sbai8
Sbai9
