# Supplementary material for: Comparative Genome Analysis of Scutellaria baicalensis and Scutellaria barbata Reveals the Evolution of Active Flavonoid Biosynthesis
Source: Genomics Proteomics Bioinformatics. 2020 Nov 4;18(3):230–40. doi: 10.1016/j.gpb.2020.06.002 (PMC7801248; doi:10.1016/j.gpb.2020.06.002)
Supplement: Supplementary Figure S11 — UPLC detection of flavonoid contents. UPLC detection of flavonoids (280 nm) in different tissues of S. baicalensis and S. barbata, including baicalein, scutellarein, wogonin, and their glycosides (baicalin, scutellarin, and wogonoside). The compound information, including detailed retention time and spectrum data, is listed in Table S12. A. Flavonoid contents of S. baicalensis. B. Flavonoid contents of S. barbata. [file mmc12.pptx]

## Slide 1
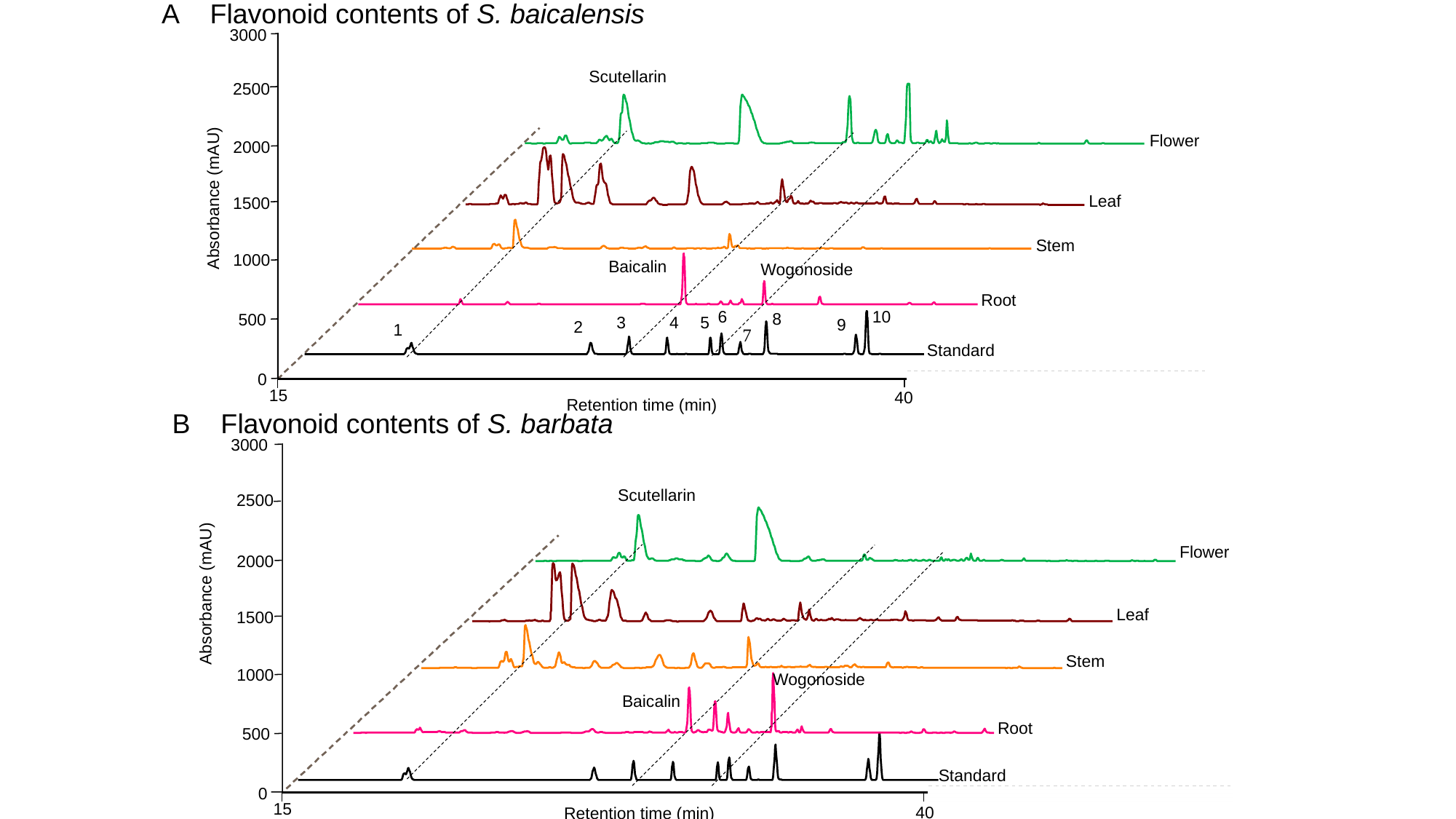

A Flavonoid contents of S. baicalensis
3000
Scutellarin
2500
Flower
2000
Absorbance (mAU)
Leaf
1500
Stem
1000
Baicalin
Wogonoside
Root
6
10
8
500
5
4
3
9
2
1
7
Standard
0
15
40
Retention time (min)
3000
Flower
Leaf
Stem
Root
Standard
Scutellarin
Absorbance (mAU)
Wogonoside
Baicalin
0
15
40
Retention time (min)
B Flavonoid contents of S. barbata
2500
2000
1500
1000
500
