# Supplementary figures and images for: Comparative Genome Analysis of Scutellaria baicalensis and Scutellaria barbata Reveals the Evolution of Active Flavonoid Biosynthesis
Source: Genomics Proteomics Bioinformatics. 2020 Nov 4;18(3):230–40. doi: 10.1016/j.gpb.2020.06.002 (PMC7801248; doi:10.1016/j.gpb.2020.06.002)

## Slide 1
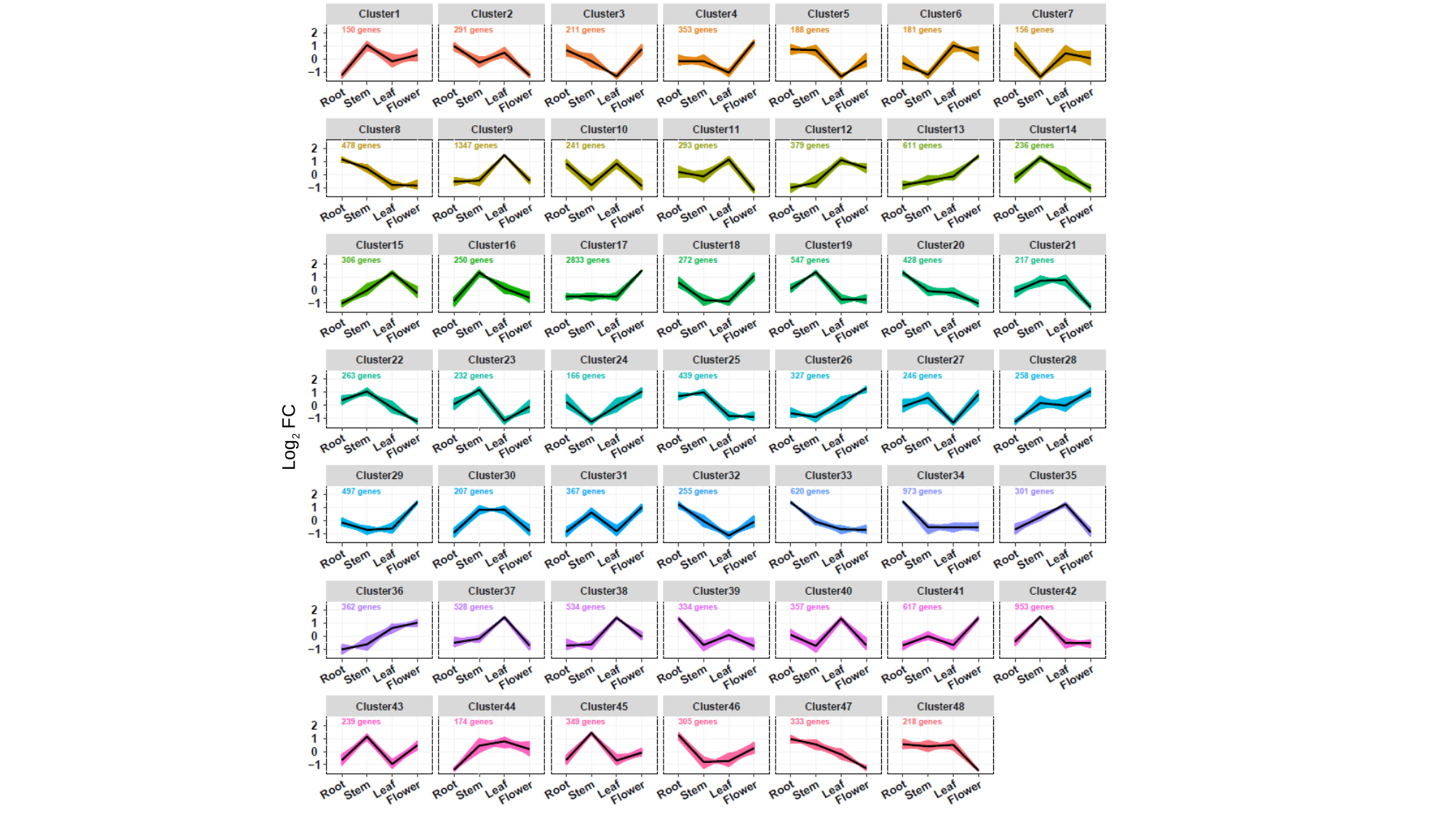

Log2 FC

Supplement: Supplementary Figure S12 — Gene expression clusters in S. baicalensis. All expressed genes were classified into 48 clusters based on k-means in different S. baicalensis tissues, namely, root, stem, leaf, and flower tissues. [file mmc13.pptx]

## Slide 1
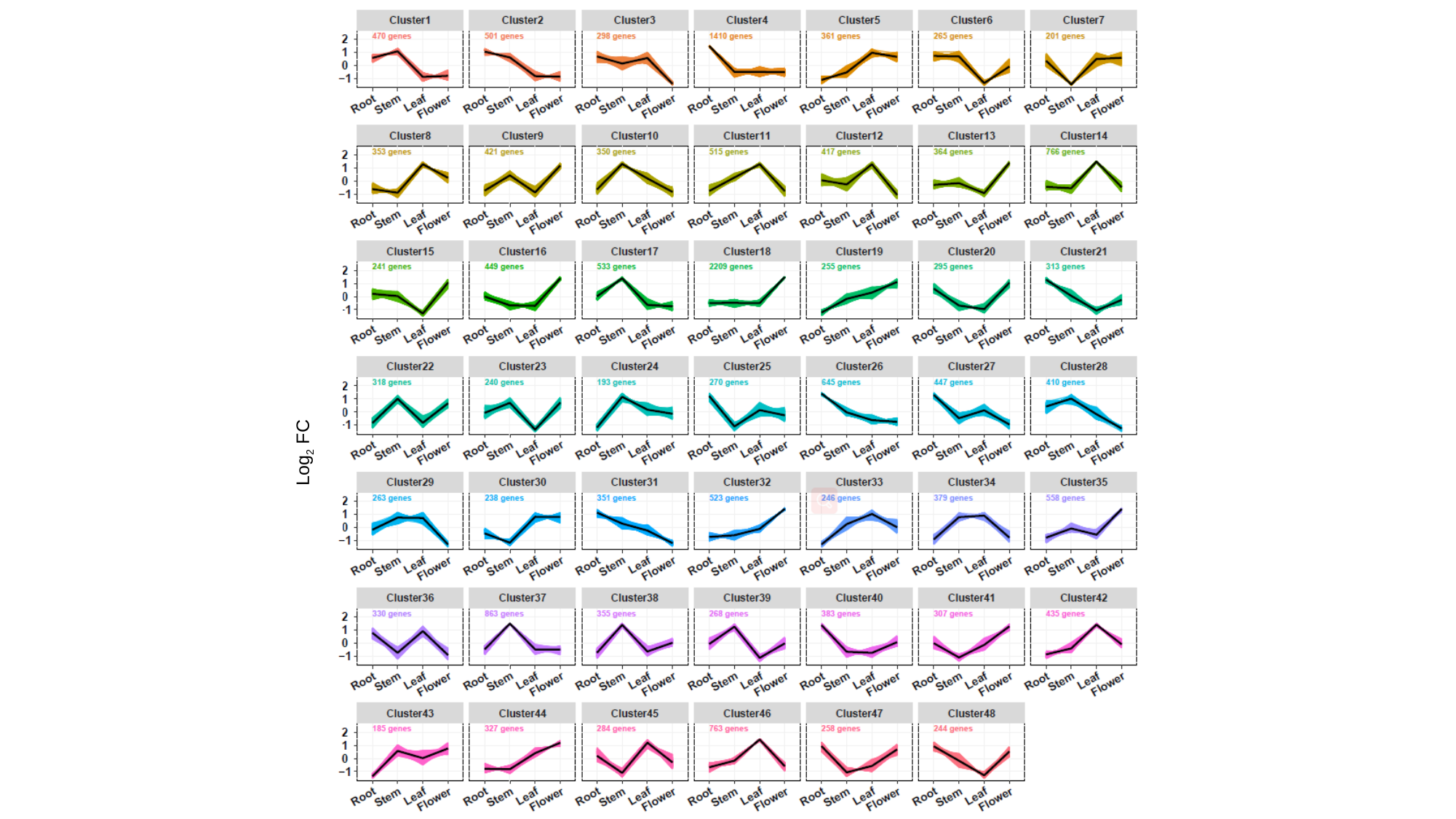

Log2 FC

Supplement: Supplementary Figure S13 — Gene expression clusters in S. barbata. All expressed genes were classified into 48 clusters based on k-means in different S. barbata tissues, namely, root, stem, leaf, and flower tissues. [file mmc14.pptx]

## Slide 1
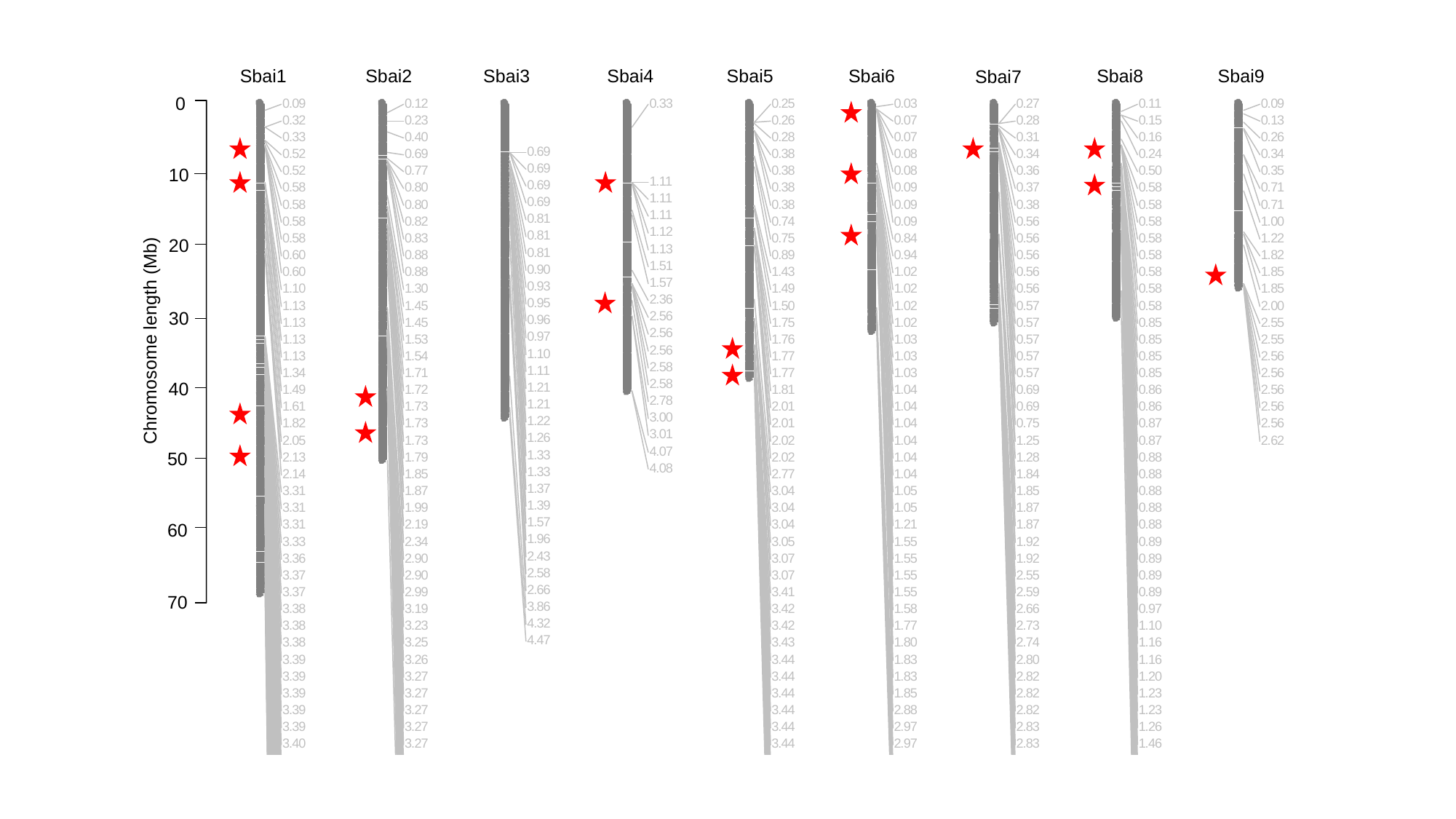

Sbai8
Sbai9
Sbai6
Sbai3
Sbai4
Sbai5
Sbai2
Sbai1
Sbai7
0
10
20
30
40
50
60
70
Chromosome length (Mb)

Supplement: Supplementary Figure S16 — Physical clusters of CYP450 genes in S. baicalensis. Regions with > 5 gene clusters per 500 kb are marked with red stars. [file mmc17.pptx]

## Slide 1
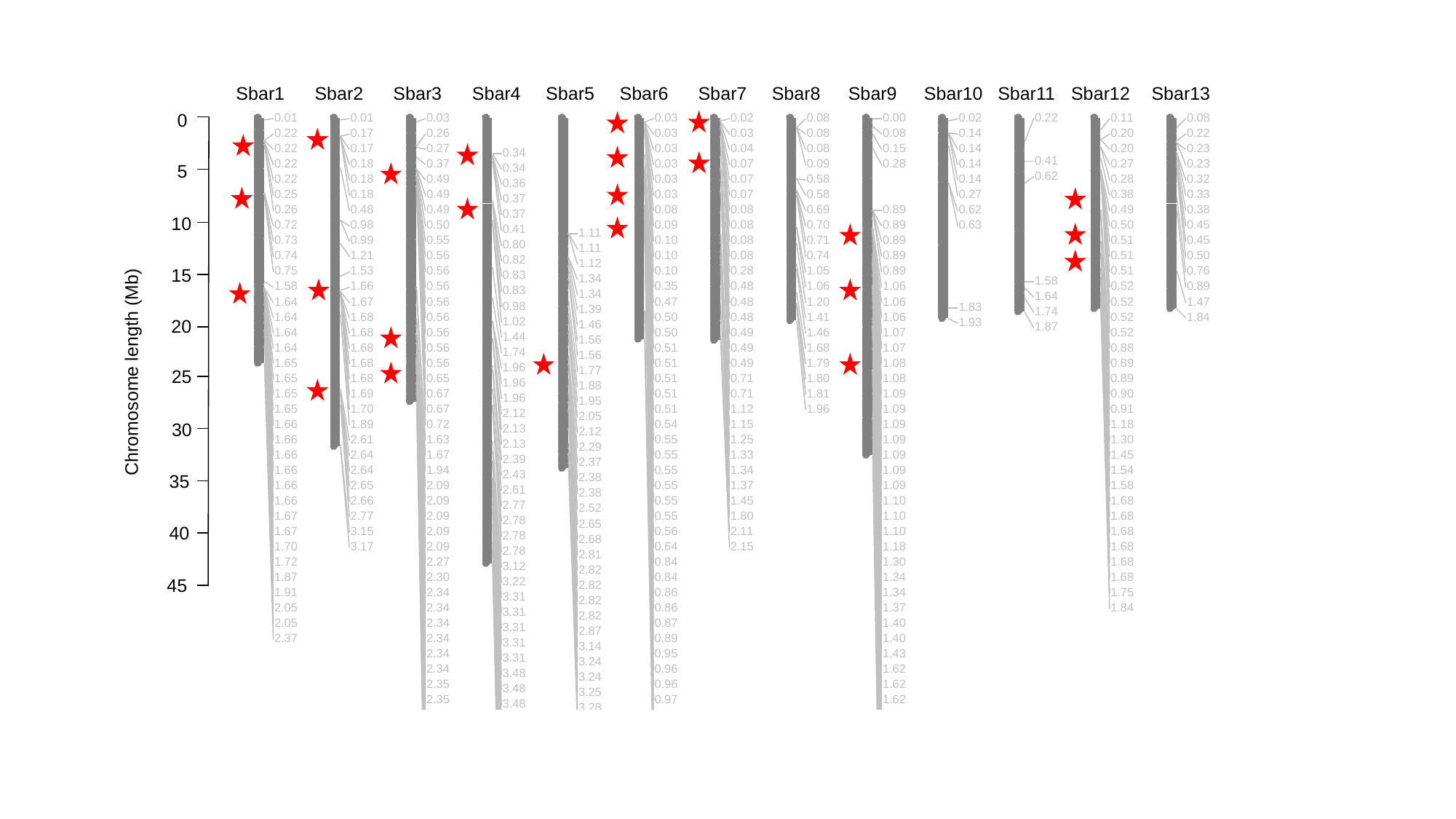

Sbar11
Sbar12
Sbar10
Sbar8
Sbar9
Sbar6
Sbar3
Sbar4
Sbar5
Sbar2
Sbar1
Sbar13
Sbar7
0
5
10
15
20
Chromosome length (Mb)
25
30
35
40
45

Supplement: Supplementary Figure S17 — Physical clusters of CYP450 genes in S. barbata. Regions with > 5 gene clusters per 500 kb are marked with red stars. [file mmc18.pptx]
