# Supplementary material for: Comparative Genome Analysis of Scutellaria baicalensis and Scutellaria barbata Reveals the Evolution of Active Flavonoid Biosynthesis
Source: Genomics Proteomics Bioinformatics. 2020 Nov 4;18(3):230–40. doi: 10.1016/j.gpb.2020.06.002 (PMC7801248; doi:10.1016/j.gpb.2020.06.002)
Supplement: Supplementary Figure S14 — Phylogenetic analysis of PAL, C4H, 4CL, and FNSII. The phylogenetic trees for PAL (A), C4H (B), 4CL (C), and FNSII (D) genes were constructed using the maximum likelihood method with bootstrap of 1000 times. [file mmc15.pptx]

## Slide 1
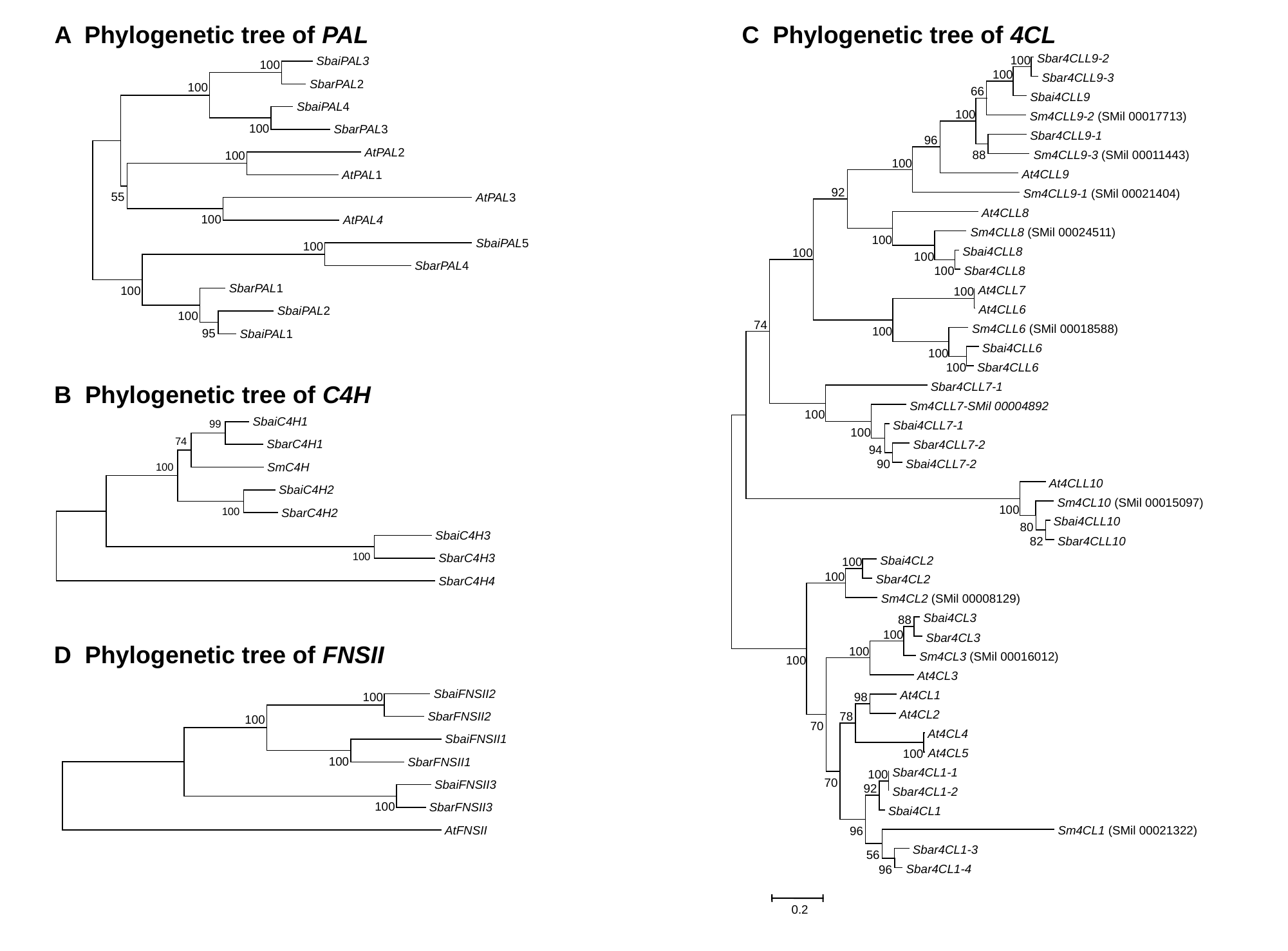

A Phylogenetic tree of PAL
C Phylogenetic tree of 4CL
 Sbar4CLL9-2
100
100
 Sbar4CLL9-3
66
 Sbai4CLL9
100
 Sm4CLL9-2 (SMil 00017713)
 Sbar4CLL9-1
96
 Sm4CLL9-3 (SMil 00011443)
88
100
 At4CLL9
92
 Sm4CLL9-1 (SMil 00021404)
 At4CLL8
 Sm4CLL8 (SMil 00024511)
100
 Sbai4CLL8
100
100
 Sbar4CLL8
100
 At4CLL7
100
 At4CLL6
74
 Sm4CLL6 (SMil 00018588)
100
 Sbai4CLL6
100
 Sbar4CLL6
100
 Sbar4CLL7-1
 Sm4CLL7-SMil 00004892
100
 Sbai4CLL7-1
100
 Sbar4CLL7-2
94
 Sbai4CLL7-2
90
 At4CLL10
 Sm4CL10 (SMil 00015097)
100
 Sbai4CLL10
80
 Sbar4CLL10
82
 Sbai4CL2
100
100
 Sbar4CL2
 Sm4CL2 (SMil 00008129)
 Sbai4CL3
88
100
 Sbar4CL3
100
 Sm4CL3 (SMil 00016012)
100
 At4CL3
 At4CL1
98
 At4CL2
78
70
 At4CL4
 At4CL5
100
 Sbar4CL1-1
100
70
92
 Sbar4CL1-2
 Sbai4CL1
 Sm4CL1 (SMil 00021322)
96
 Sbar4CL1-3
56
 Sbar4CL1-4
96
0.2
 SbaiPAL3
100
 SbarPAL2
100
 SbaiPAL4
100
 SbarPAL3
 AtPAL2
100
 AtPAL1
55
 AtPAL3
100
 AtPAL4
 SbaiPAL5
100
 SbarPAL4
 SbarPAL1
100
 SbaiPAL2
100
95
 SbaiPAL1
B Phylogenetic tree of C4H
 SbaiC4H1
99
74
 SbarC4H1
 SmC4H
100
 SbaiC4H2
100
 SbarC4H2
 SbaiC4H3
100
 SbarC4H3
 SbarC4H4
D Phylogenetic tree of FNSII
 SbaiFNSII2
100
 SbarFNSII2
100
 SbaiFNSII1
100
 SbarFNSII1
 SbaiFNSII3
100
 SbarFNSII3
 AtFNSII
