# Supplementary material for: Comparative Genome Analysis of Scutellaria baicalensis and Scutellaria barbata Reveals the Evolution of Active Flavonoid Biosynthesis
Source: Genomics Proteomics Bioinformatics. 2020 Nov 4;18(3):230–40. doi: 10.1016/j.gpb.2020.06.002 (PMC7801248; doi:10.1016/j.gpb.2020.06.002)
Supplement: Supplementary Figure S15 — Potential downstream biosynthetic pathway. The biosynthesis of baicalein, scutellarein, wogonin, and their glycosides (baicalin, scutellarin, and wogonoside). A. Chrysin as substrate. F6H catalyzes chrysin to produce baicalein. F8H transforms chrysin to norwogonin, and F8OMT further catalyzes norwogonin to wogonin. Then, UBGAT perform the transfer of glucuronic acid to the 7-OH of baicalein, norwogonin, and wogonin. B. Apigenin as substrate. F6H transforms apigenin to scutellarein, then UBGAT catalyzes the transfer of glucuronic acid to the 7-OH of scutellarein to produce scutellarin. F6H, flavone 6-hydroxylase; F8H, flavone 8-hydroxylase; F8OMT, flavone 8-O-methyltransferases; UGAT, UDP- glucuronosyltransferase. [file mmc16.pptx]

## Slide 1
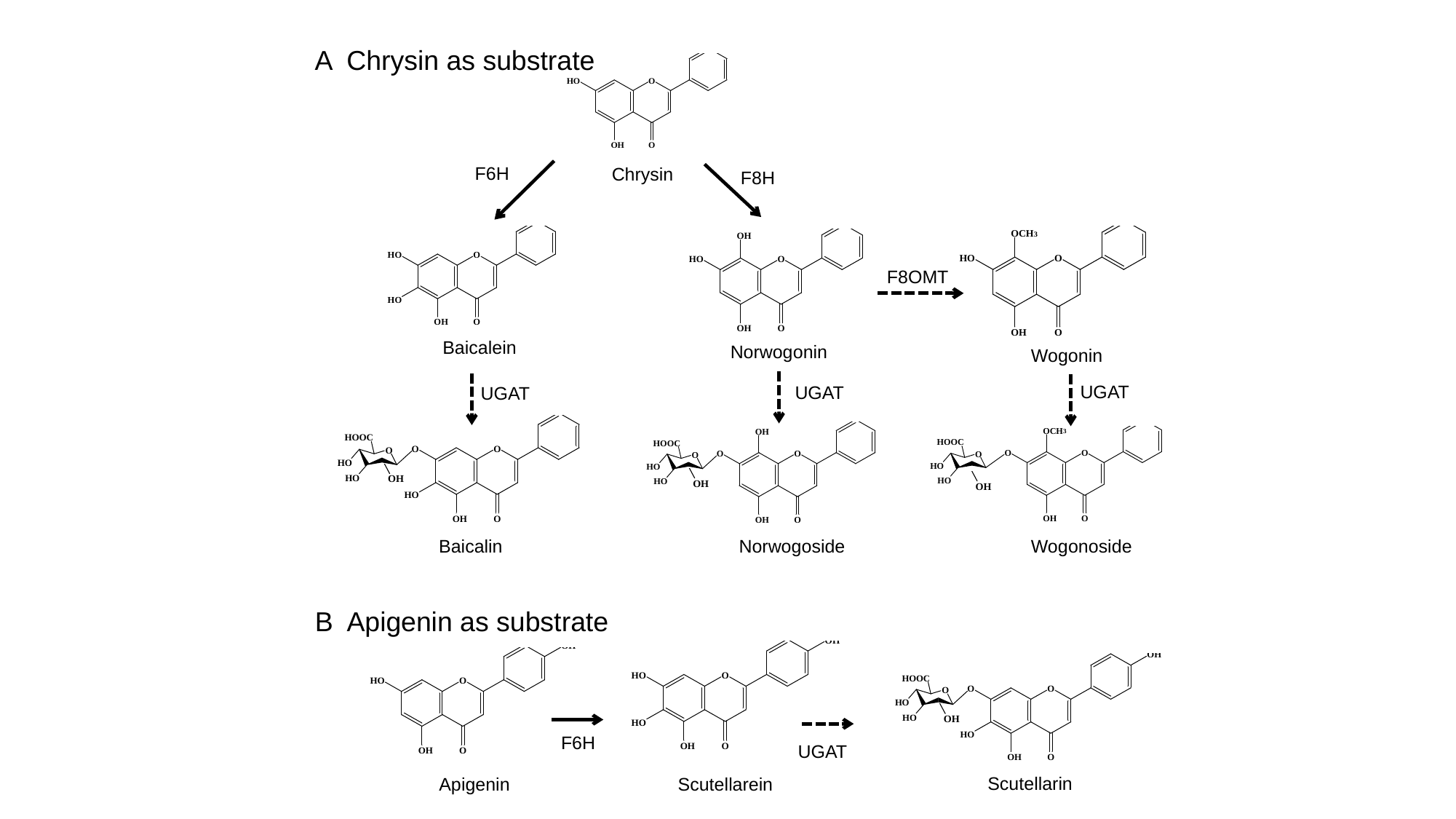

A Chrysin as substrate
F6H
Chrysin
F8H
F8OMT
Baicalein
Norwogonin
Wogonin
UGAT
UGAT
UGAT
Wogonoside
Baicalin
Norwogoside
B Apigenin as substrate
F6H
UGAT
Scutellarin
Apigenin
Scutellarein
