# Supplementary material for: Comparative Genome Analysis of Scutellaria baicalensis and Scutellaria barbata Reveals the Evolution of Active Flavonoid Biosynthesis
Source: Genomics Proteomics Bioinformatics. 2020 Nov 4;18(3):230–40. doi: 10.1016/j.gpb.2020.06.002 (PMC7801248; doi:10.1016/j.gpb.2020.06.002)
Supplement: Supplementary Figure S18 — Phylogenetic analysis of CYP82D, CYP93B, and CYP73A members. The phylogenetic trees of CYP82D, CYP93B, and CYP73A members from S. baicalensis and S. barbata were constructed using the maximum likelihood method with bootstrap of 1000 times. [file mmc19.pptx]

## Slide 1
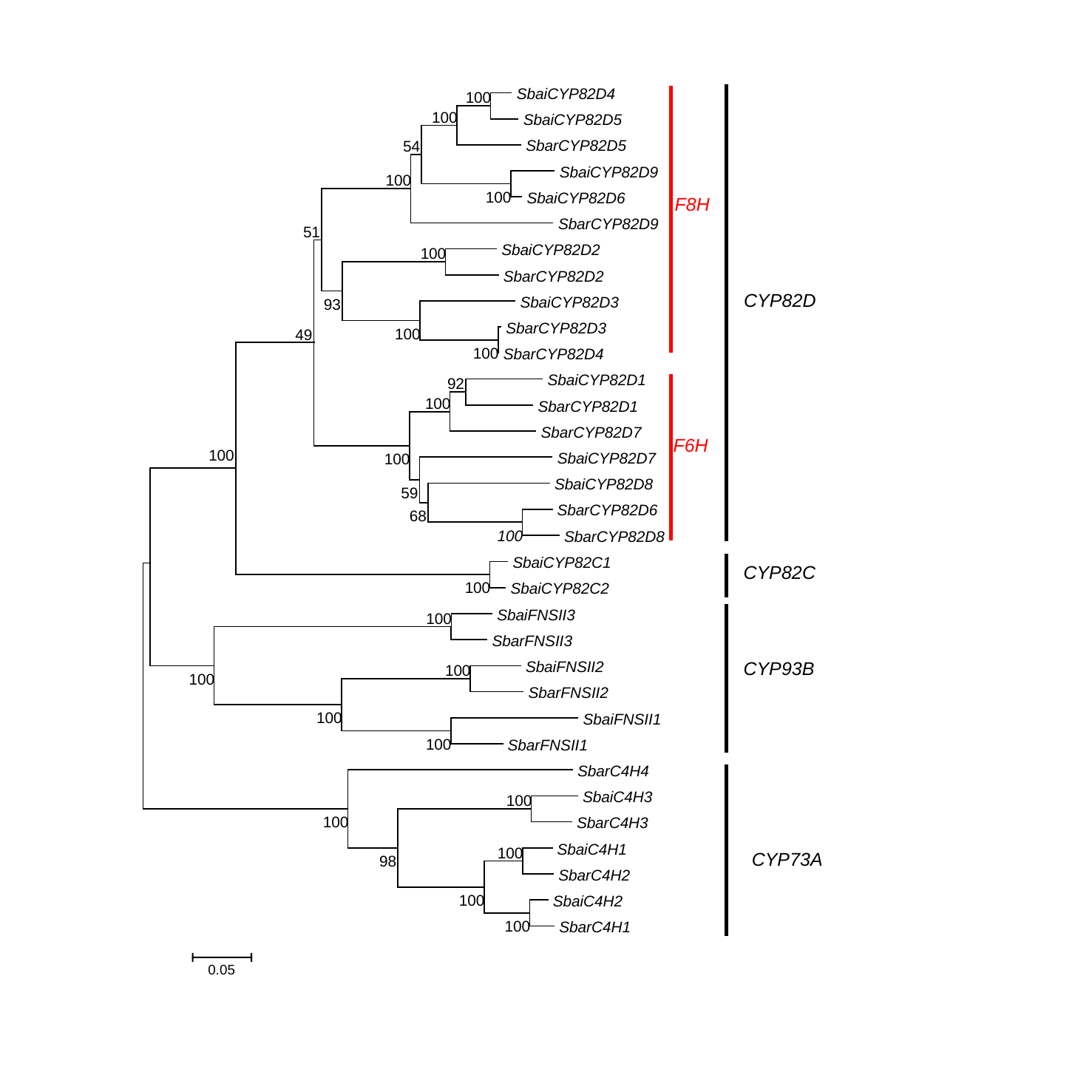

SbaiCYP82D4
100
100
 SbaiCYP82D5
 SbarCYP82D5
54
 SbaiCYP82D9
100
100
 SbaiCYP82D6
F8H
 SbarCYP82D9
51
 SbaiCYP82D2
100
 SbarCYP82D2
CYP82D
 SbaiCYP82D3
93
 SbarCYP82D3
100
49
100
 SbarCYP82D4
 SbaiCYP82D1
92
100
 SbarCYP82D1
 SbarCYP82D7
F6H
100
 SbaiCYP82D7
100
 SbaiCYP82D8
59
 SbarCYP82D6
68
100
 SbarCYP82D8
 SbaiCYP82C1
CYP82C
100
 SbaiCYP82C2
 SbaiFNSII3
100
 SbarFNSII3
 SbaiFNSII2
CYP93B
100
100
 SbarFNSII2
100
 SbaiFNSII1
100
 SbarFNSII1
 SbarC4H4
 SbaiC4H3
100
100
 SbarC4H3
 SbaiC4H1
100
CYP73A
98
 SbarC4H2
100
 SbaiC4H2
100
 SbarC4H1
0.05
