# Supplementary material for: Comparative Genome Analysis of Scutellaria baicalensis and Scutellaria barbata Reveals the Evolution of Active Flavonoid Biosynthesis
Source: Genomics Proteomics Bioinformatics. 2020 Nov 4;18(3):230–40. doi: 10.1016/j.gpb.2020.06.002 (PMC7801248; doi:10.1016/j.gpb.2020.06.002)
Supplement: Supplementary Table S1 — Statistics of sequencingdata. [file mmc20.docx]

**Table S1 Statistics of sequencing data**

| **Parameter** | ***S. baicalensis* (ONT)** | ***S. barbata* (SMRT)** |
| --- | --- | --- |
| No. of raw reads | 3,193,420 | 8,423,154 |
| Size of raw data (bp) | 52,035,200,672 | 51,672,515,843 |
| N50 of raw reads (bp) | 16,324 | 9843 |
| No. of filtered reads | 2,761,758 | 7,811,959 |
| Size of filtered data (bp) | 47,594,396,058 | 483,96,743,827 |
| N50 of filtered reads (bp) | 23,750 | 10,206 |
| No. of corrected reads using Canu | 567,210 | 1,154,193 |
| Size of corrected data (bp) | 20,220,782,775 | 18,047,628,482 |
| N50 of corrected reads (bp) | 35,491 | 15,286 |

*Note*: ONT, Oxford Nanopore Technologies; SMRT, single-molecule sequencing in real time.
