# Supplementary material for: Comparative Genome Analysis of Scutellaria baicalensis and Scutellaria barbata Reveals the Evolution of Active Flavonoid Biosynthesis
Source: Genomics Proteomics Bioinformatics. 2020 Nov 4;18(3):230–40. doi: 10.1016/j.gpb.2020.06.002 (PMC7801248; doi:10.1016/j.gpb.2020.06.002)
Supplement: Supplementary Table S2 — Statistics of Scutellaria genomeassembly. [file mmc21.docx]

**Table S2 Statistics of** ***Scutellaria* genome assembly**

| **Parameter** | ***S. baicalensis*** | ***S. barbata*** |
| --- | --- | --- |
| Predicted genome size (bp) | 441,861,814 | 404,561,255 |
| Assembled genome size (bp) | 376,971,275 | 352,950,734 |
| Percentage of assembled genome of the predicted genome | 85.3% | 87.2% |
| Contig N50 (bp) | 2,102,880 | 2,496,969 |
| No. of chromosomes assembled | 9 | 13 |
| Size of chromosomal genome (bp) | 376,437,573 | 348,847,092 |
| Scaffold N50 (bp) | 40,790,749 | 23,709,848 |
| DNA mapping rate (Illumina reads) | 88.72% | 86.13% |
| RNA mapping rate (Illumina reads) | 82.97% | 86.56% |
| BUSCO | C: 91.5%, F: 2.6%, M: 5.9%, n: 1440 | C: 93.0%, F: 2.0%, M: 5.0%, n: 1440 |

*Note*: For BUSCO categories, C, F, and M indicate the percentage of complete, fragmented, and missing BUSCOs, respectively, while n indicates the total BUSCO groups searched.
