# Supplementary material for: Comparative Genome Analysis of Scutellaria baicalensis and Scutellaria barbata Reveals the Evolution of Active Flavonoid Biosynthesis
Source: Genomics Proteomics Bioinformatics. 2020 Nov 4;18(3):230–40. doi: 10.1016/j.gpb.2020.06.002 (PMC7801248; doi:10.1016/j.gpb.2020.06.002)
Supplement: Supplementary Table S3 — Statistics of genomeannotations. [file mmc22.docx]

**Table S3 Statistics of genome annotations**

| **Annotation** | ***S. baicalensis*** | ***S. barbata*** | ***S. miltiorrhiza*** |
| --- | --- | --- | --- |
| No. of predicted transcripts and proteins | 33,414 | 41,697 | 30,478 |
| Average gene length (bp) | 4553 | 2257 | 2826 |
| Average CDS length (bp) | 1130 | 1137 | 1173 |
| No. of exons | 161,060 | 182,344 | 164,031 |
| Average exon length (bp) | 234 | 260 | 228 |
| Average intron length (bp) | 296 | 337 | 268 |
| GC content of transcripts (%) | 44.92 | 45.34 | 47.96 |
| Percentage of whole gene length in genome (%) | 10.01 | 13.43 | 6.65 |
| BUSCO | C:87.3%, F:5.9%, M:6.8% | C:89.7%, F:4.6%, M:5.7% | C:84.6%, F:6.5%, M:8.9%, n:1375 |
| Masked repeat sequence length (bp) | 208,004,279 | 188,790,851 | 292,797,272 |
| Percentage of repeat sequences in genome (%) | 55.17 | 53.49 | 54.44 |
