# Supplementary material for: Comparative Genome Analysis of Scutellaria baicalensis and Scutellaria barbata Reveals the Evolution of Active Flavonoid Biosynthesis
Source: Genomics Proteomics Bioinformatics. 2020 Nov 4;18(3):230–40. doi: 10.1016/j.gpb.2020.06.002 (PMC7801248; doi:10.1016/j.gpb.2020.06.002)
Supplement: Supplementary Table S5 — Annotation of S. baicalensisTEs. [file mmc24.docx]

**Table S5 Annotation of *S. baicalensis* TEs**

| **Repeat class** | **No. of elements** | **Length occupied (bp)** | **Percentage in the whole genome** |
| --- | --- | --- | --- |
| **RNA elements** | **188,820** | **123,378,922** | **32.73 %** |
| LINE | 7183 | 3,023,969 | 0.80 % |
| SINE | 2604 | 52,3641 | 0.14 % |
| *Gypsy* | 36,704 | 31,904,065 | 8.46 % |
| *Copia* | 43,124 | 43,848,306 | 11.63 % |
| Others | 99,175 | 44,078,941 | 11.7 % |
| **DNA elements** | **90,835** | **39,583,481** | **10.50 %** |
| **Unclassified TEs** | **133,416** | **39,607,418** | **10.51 %** |
| **Others** | **183,732** | **5,434,458** | **1.44%** |
| **All repeats** | **596,803** | **208,004,279** | 55.18 % |

*Note*: TE, transposable element; LTR, long terminal repeat; LINE, long interspersed nuclear element; SINE, short interspersed nuclear element.
