# Supplementary material for: Comparative Genome Analysis of Scutellaria baicalensis and Scutellaria barbata Reveals the Evolution of Active Flavonoid Biosynthesis
Source: Genomics Proteomics Bioinformatics. 2020 Nov 4;18(3):230–40. doi: 10.1016/j.gpb.2020.06.002 (PMC7801248; doi:10.1016/j.gpb.2020.06.002)
Supplement: Supplementary Table S6 — Annotation of S. barbataTEs. [file mmc25.docx]

**Table S6 Annotation of *S. barbata* TEs**

| **Repeat class** | **No. of elements** | **Length occupied (bp)** | **Percentage in the whole genome** |
| --- | --- | --- | --- |
| **RNA elements** | **110,236** | **115,578,006** | **32.75 %** |
| LINE | 4080 | 2,157,913 | 0.61 % |
| SINE | 889 | 304,212 | 0.09 % |
| *Gypsy* | 36,745 | 28,270,899 | 8.01 % |
| *Copia* | 35,064 | 62,130,229 | 17.60 % |
| Others | 33,458 | 22,714,753 | 6.44 % |
| **DNA elements** | **66,220** | **30,502,000** | **8.64 %** |
| **Unclassified TEs** | **127,635** | **36,877,920** | **10.45%** |
| **Others** | **177,940** | **5,832,925** | **1.65%** |
| **All repeats** | **482,031** | **188,790,851** | 53.49 % |

*Note*: TE, transposable element; LTR, long terminal repeat; LINE, long interspersed nuclear element; SINE, short interspersed nuclear element.
