# Supplementary material for: Comparative Genome Analysis of Scutellaria baicalensis and Scutellaria barbata Reveals the Evolution of Active Flavonoid Biosynthesis
Source: Genomics Proteomics Bioinformatics. 2020 Nov 4;18(3):230–40. doi: 10.1016/j.gpb.2020.06.002 (PMC7801248; doi:10.1016/j.gpb.2020.06.002)
Supplement: Supplementary Table S7 — Summary of intact LTRretrotransposons. [file mmc26.docx]

**Table S7 Summary of intact LTR retrotransposons**

| **Parameter** | ***S. baicalensis*** | ***S. barbata*** |
| --- | --- | --- |
| No. of intact LTRs | 1225 | 1654 |
| Average inserting time of all intact LTRs (MYA) | 1.41 | 0.88 |
| *Gypsy* | 342 | 310 |
| Maximal insertion time of *Gypsy* (MYA) | 4.14 | 4.39 |
| Minimal insertion time of *Gypsy* (MYA) | 0.072 | 0.015 |
| Average insertion time of *Gypsy* (MYA) | 1.42 | 0.96 |
| *Copia* | 354 | 618 |
| Maximal insertion time of *Copia* (MYA) | 6.92 | 3.87 |
| Minimal insertion time of *Copia* (MYA) | 0.14 | 0.047 |
| Average insertion time of *Copia* (MYA) | 1.55 | 0.90 |

*Note*: LTR, long terminal repeat; MYA, million years ago.
