# Supplementary material for: Comparative Genome Analysis of Scutellaria baicalensis and Scutellaria barbata Reveals the Evolution of Active Flavonoid Biosynthesis
Source: Genomics Proteomics Bioinformatics. 2020 Nov 4;18(3):230–40. doi: 10.1016/j.gpb.2020.06.002 (PMC7801248; doi:10.1016/j.gpb.2020.06.002)
Supplement: Supplementary Table S8 — Annotation of S. baicalensis and S. barbata rRNAgenes. [file mmc27.docx]

**Table S8 Annotation of *S. baicalensis* and *S. barbata* rRNA genes**

|  | ***S. baicalensis*** | ***S. barbata*** |
| --- | --- | --- |
| No. of rRNA genes | 813 | 210 |
| No. of 8S rRNA genes | 785 | 189 |
| No. of 18S rRNA genes | 16 | 8 |
| No. of 28S rRNA genes | 12 | 13 |
