# Supplementary material for: Comparative Genome Analysis of Scutellaria baicalensis and Scutellaria barbata Reveals the Evolution of Active Flavonoid Biosynthesis
Source: Genomics Proteomics Bioinformatics. 2020 Nov 4;18(3):230–40. doi: 10.1016/j.gpb.2020.06.002 (PMC7801248; doi:10.1016/j.gpb.2020.06.002)
Supplement: Supplementary Table S9 — Identification ofSSRs. [file mmc28.docx]

**Table S9 Identification of SSRs**

| **Type** | **Unit size** | **Cut-off value for No. of repeats** | **No. of SSRs in *S. baicalensis*** | **No. of SSRs in *S. barbata*** |
| --- | --- | --- | --- | --- |
| Monomer | 1 | 10 | 73,270 | 93,162 |
| Dimer | 2 | 6 | 58,417 | 42,280 |
| Trimer | 3 | 5 | 9876 | 10,742 |
| Tetramer | 4 | 5 | 740 | 1160 |
| Pentamer | 5 | 5 | 373 | 213 |
| Hexamer | 6 | 5 | 275 | 148 |
| Total |  |  | 142,951 | 147,705 |

*Note*: SSR, single sequence repeat.
