# Supplementary material for: Comparative Genome Analysis of Scutellaria baicalensis and Scutellaria barbata Reveals the Evolution of Active Flavonoid Biosynthesis
Source: Genomics Proteomics Bioinformatics. 2020 Nov 4;18(3):230–40. doi: 10.1016/j.gpb.2020.06.002 (PMC7801248; doi:10.1016/j.gpb.2020.06.002)
Supplement: Supplementary Table S10 — Gene family expansion andcontraction. [file mmc29.docx]

**Table S10 Gene family expansion and contraction**

| **Phylogenetic tree node** | **Expansion** | **Remain** | **Contraction** |
| --- | --- | --- | --- |
| 0 | 2176 | 16,237 | 964 |
| 2 | 965 | 15,169 | 3344 |
| 1 | 1639 | 10,281 | 7552 |
| 13 | 475 | 16,475 | 2527 |
| 4 | 1180 | 16,697 | 1599 |
| 6 | 1853 | 15,993 | 1632 |
| 5 | 693 | 17,055 | 1727 |
| 8 | 2543 | 12,664 | 4271 |
| 7 | 109 | 18,813 | 550 |
| 10 | 5117 | 11,055 | 3305 |
| 9 | 1145 | 16,699 | 1629 |
| 12 | 6388 | 9067 | 4023 |
| 11 | 856 | 17,742 | 878 |
| 17 | 47 | 16,797 | 2634 |
| 14 | 2142 | 13,014 | 4320 |
| 16 | 5934 | 11,843 | 1699 |
| 15 | 43 | 19,417 | 11 |
| 18 | 1306 | 13,251 | 4921 |
| 3 | 143 | 19,125 | 207 |
| 20 | 1104 | 7030 | 11,344 |

*Note*: The node numbers are shown in Figure S8. Remain means that no gene family expansion or contraction occur.
