# Supplementary material for: Comparative Genome Analysis of Scutellaria baicalensis and Scutellaria barbata Reveals the Evolution of Active Flavonoid Biosynthesis
Source: Genomics Proteomics Bioinformatics. 2020 Nov 4;18(3):230–40. doi: 10.1016/j.gpb.2020.06.002 (PMC7801248; doi:10.1016/j.gpb.2020.06.002)
Supplement: Supplementary Table S11 — The Ks value and divergence time of paralogous or orthologous genepairs. [file mmc30.docx]

**Table S11 The *K*_S_ value and divergence time of paralogous or orthologous gene pairs**

| **Species 1** | **Species 2** | **No. of orthologous or paralogous gene pairs** | ***K*_S_ peak** | **Divergence time for speciation (MYA)** | **WGD time (MYA)** | **Synonymous substitutions per site per MYA** |
| --- | --- | --- | --- | --- | --- | --- |
| *S. baicalensis* | *S. baicalensis* | 5978 | 0.886467515 | N/A | 60.71031093 | N/A |
| *S. barbata* | *S. barbata* | 5366 | 0.862984932 | N/A | 59.10209078 | N/A |
| *S. miltiorrhiza* | *S. miltiorrhiza* | 5764 | 1.021492368 | N/A | 69.95757681 | N/A |
| *S. indicum* | *S. indicum* | 6702 | 0.675124266 | N/A | 46.23632949 | N/A |
| *V. vinifera* | *V. vinifera* | 1993 | 1.203482387 | N/A | N/A | N/A |
| *S. baicalensis* | *S. miltiorrhiza* | 11,028 | 0.598805871 | 41.009614 | N/A | 0.007300799 |
| *S. baicalensis* | *S. indicum* | 11,712 | 0.604676517 | 49.897716 | N/A | 0.006059161 |
| *S. baicalensis* | *V. vinifera* | 6098 | 1.526367906 | 115.832779 | N/A | 0.00658867 |
| *S. barbata* | *S. miltiorrhiza* | 11,123 | 0.581193933 | 41.009614 | N/A | 0.00708607 |
| *S. barbata* | *S. indicum* | 11,936 | 0.557711350 | 49.897716 | N/A | 0.005588546 |
| *S. barbata* | *V. vinifera* | 6282 | 1.514626614 | 115.832779 | N/A | 0.006537988 |
| *S. baicalensis* | *S. barbata* | 16,204 | 0.16437802 | 13.281676 | N/A | 0.006188151 |

*Note*: *K***_S_**, synonymous substitution rate; MYA, million years ago; WGD, whole-genome duplication; N/A, not applicable.
