# Supplementary material for: Comparative Genome Analysis of Scutellaria baicalensis and Scutellaria barbata Reveals the Evolution of Active Flavonoid Biosynthesis
Source: Genomics Proteomics Bioinformatics. 2020 Nov 4;18(3):230–40. doi: 10.1016/j.gpb.2020.06.002 (PMC7801248; doi:10.1016/j.gpb.2020.06.002)
Supplement: Supplementary Table S12 — Compound information ofUPLC detection. [file mmc31.docx]

**Table S12 Compound information of UPLC detection**

|  | **Compound** | **Retention time (min)** | **λmax (nm)** | **MW** |
| --- | --- | --- | --- | --- |
| 1 | Scutellarin | 19.303 | 282.50 | 462.37 |
| 2 | Scutellarein | 26.553 | 283.20 | 286.24 |
| 3 | Baicalin | 28.097 | 277.50 | 446.37 |
| 4 | Norwogoside | 29.637 | 279.97 | 446.36 |
| 5 | Wogonoside | 31.383 | 274.32 | 460.39 |
| 6 | Apigenin | 31.827 | 267.26 | 270.24 |
| 7 | Norwogonin | 32.590 | 280.41 | 270.24 |
| 8 | Baicalein | 33.637 | 275.72 | 270.24 |
| 9 | Wogonin | 37.263 | 275.23 | 284.27 |
| 10 | Chrysin | 37.697 | 265.65 | 254.24 |

*Note*: MW, molecular weight. The peaks are numbered and shown in Figure S11.
