# Supplementary material for: Comparative Genome Analysis of Scutellaria baicalensis and Scutellaria barbata Reveals the Evolution of Active Flavonoid Biosynthesis
Source: Genomics Proteomics Bioinformatics. 2020 Nov 4;18(3):230–40. doi: 10.1016/j.gpb.2020.06.002 (PMC7801248; doi:10.1016/j.gpb.2020.06.002)
Supplement: Supplementary Table S16 — Expression of chrysin and apigenin biosynthetic genes inS. barbata. [file mmc35.docx]

**Table S16 Expression of chrysin and apigenin biosynthetic genes in *S. barbata***

| **Gene name** | **Gene ID** | **Root** | **Stem** | **Leaf** | **Flower** |
| --- | --- | --- | --- | --- | --- |
| *SbarPAL1* | Sbar5A218T212 | 18.55 | 68.76 | 16.08 | 62.55 |
| *SbarPAL2* | Sbar4A4T132 | 147.95 | 418.11 | 251.03 | 222.89 |
| *SbarPAL3* | Sbar11A170T161 | 83.38 | 84.76 | 61.14 | 19.06 |
| *SbarPAL4* | Sbar10A150T96 | 6.81 | 2.81 | 0.78 | 0.11 |
|  |  |  |  |  |  |
| *Sbar4CL1-1* | Sbar4C297T22 | 1.16 | 1.78 | 0.98 | 0.53 |
| *Sbar4CL1-2* | Sbar4C298T24 | 7.10 | 8.72 | 2.64 | 2.11 |
| *Sbar4CL1-3* | Sbar3A77T120 | 0.63 | 0.59 | 0.01 | 0.01 |
| *Sbar4CL1-4* | Sbar9A326T113 | 25.62 | 5.18 | 0.85 | 1.68 |
| *Sbar4CL2* | Sbar5A256T179 | 67.84 | 29.78 | 22.22 | 159.44 |
| *Sbar4CL3* | Sbar4A380T150 | 223.87 | 243.07 | 186.02 | 62.82 |
| *Sbar4CLL6* | Sbar9A255T177 | 0.26 | 5.40 | 63.86 | 10.23 |
| *Sbar4CLL7-1* | Sbar13A3T207 | 32.03 | 23.25 | 36.33 | 35.24 |
| *Sbar4CLL7-2* | Sbar3A216T75 | 0.00 | 0.30 | 0.29 | 3.34 |
| *Sbar4CLL8* | Sbar6A181T46 | 40.42 | 143.13 | 275.04 | 14.78 |
| *Sbar4CLL9-1* | Sbar4A312T146 | 0.37 | 0.24 | 0.29 | 3.03 |
| *Sbar4CLL9-2* | Sbar9C331T22 | 1.76 | 0.49 | 13.25 | 10.37 |
| *Sbar4CLL9-3* | Sbar9A331T186 | 0.02 | 5.62 | 175.03 | 3.84 |
| *Sbar4CLL10* | Sbar5A154T110 | 602.54 | 392.44 | 517.89 | 49.31 |
|  |  |  |  |  |  |
| *SbarCHS1* | Sbar2C282T9 | 3098.44 | 5287.19 | 3673.26 | 1562.77 |
| *SbarCHS2* | Sbar5A272T111 | 0.84 | 3.30 | 0.50 | 3.20 |
| *SbarCHS3* | Sbar5A316T184 | 0.12 | 0.77 | 0.23 | 34.47 |
|  |  |  |  |  |  |
| *SbarCHI* | Sbar1A223T166 | 511.65 | 538.07 | 315.10 | 330.28 |
|  |  |  |  |  |  |
| *SbarFNSII1* | Sbar13C32T13 | 10.55 | 87.50 | 78.56 | 340.41 |
| *SbarFNSII2* | Sbar13A32T113 | 215.62 | 28.64 | 0.65 | 0.29 |
| *SbarFNSII3* | Sbar4C196T19 | 9.31 | 0.48 | 0.13 | 0.19 |
|  |  |  |  |  |  |
| *SbarC4H1* | Sbar9A209T130 | 110.87 | 389.91 | 195.03 | 168.72 |
| *SbarC4H2* | Sbar13C44T18 | 126.39 | 96.22 | 21.43 | 150.01 |
| *SbarC4H3* | Sbar9A209T128 | 0.30 | 0.91 | 2.53 | 0.15 |
| *SbarC4H4* | Sbar4A98T57 | 1.17 | 0.37 | 0.00 | 0.00 |

*Note*: Gene expression in different tissues is detected by RNA-seq and presented as FPKM values.
