# Supplementary material for: Comparative Genome Analysis of Scutellaria baicalensis and Scutellaria barbata Reveals the Evolution of Active Flavonoid Biosynthesis
Source: Genomics Proteomics Bioinformatics. 2020 Nov 4;18(3):230–40. doi: 10.1016/j.gpb.2020.06.002 (PMC7801248; doi:10.1016/j.gpb.2020.06.002)
Supplement: Supplementary Table S19 — Ks values of gene pairs related to flavonebiosynthesis. [file mmc38.docx]

**Table S19 *Ks* values of gene pairs related to flavone biosynthesis**

| **Species** | **Paralogous gene pair** | ***Ks*** |
| --- | --- | --- |
| *S. baicalensis* | *SbaiPAL1 – SbaiPAL2* | 0.0440141 |
|  | *SbaiPAL3 – SbaiPAL4* | 1.82591 |
|  | *Sbai4CL2 – Sbai4CL3* | 3.17105 |
|  | *Sbai4CLL7-1–Sbai4CLL7-2* | 0.251031 |
|  | *SbaiCHS4 – SbaiCHS3* | 0.0806434 |
|  | *SbaiCHS3 – SbaiCHS5* | 0.00586652 |
|  | *SbaiCHS5 – SbaiCHS4* | 0.0825493 |
|  | *SbaiCHS5 – SbaiCHS1* | 0.379022 |
|  | *SbaiCHS1 – SbaiCHS4* | 0.368591 |
|  | *SbaiCHS7 – SbaiCHS8* | 3.13754 |
|  | *SbaiFNS1 – SbaiFNS2* | 1.94371 |
|  | *SbaiC4H1 – SbaiC4H2* | 1.69773 |
|  | *SbaiCYP82D8 – SaiCYP82D1* | 0.87768 |
|  | *SbaiCYP82D8 – SbaiCYP82D7* | 0.634991 |
|  | *SbaiCYP82D2 – SbaiCYP82D3* | 0.942118 |
|  | *SbaiCYP82D4 – SbaiCYP82D5* | 0.0803808 |
|  | *SbaiCYP82D6 – SbaiCYP82D9* | 0.0707554 |
| *S. barbata* | *SbarPAL2 – SbarPAL3* | 1.06785 |
|  | *Sbar4CL1-1 – Sbar4CL1-2* | 0 |
|  | *Sbar4CL1-3 – Sbar4CL1-4* | 0.177134 |
|  | *Sbar4CLL9-2 –Sbar4CLL9-3* | 0.0651871 |
|  | *SbarCHS2 – SbarCHS3* | 3.07387 |
|  | *SbarFNS1 – SbarFNS2* | 1.14678 |
|  | *SbarC4H1 – SbarC4H2* | 1.38873 |
|  | *SbarCYP82D8 – SbarCYP82D1* | 0.763061 |
|  | *SbarCYP82D8 – SbarCYP82D7* | 0.629526 |
|  | *SbarCYP82D8 – SbarCYP82D6* | 0.0559024 |
|  | *SbarCYP82D2 – SbarCYP82D3* | 1.02687 |
|  | *SbarCYP82D3 – SbarCYP82D4* | 0.0115003 |
|  | *SbarCYP82D5 – SbarCYP82D9* | 0.335215 |

*Note*: *Ks*, synonymous substitution rate.
